# A Multi-scale Computational Platform to Mechanistically Assess the Effect of Genetic Variation on Drug Responses in Human Erythrocyte Metabolism

**DOI:** 10.1371/journal.pcbi.1005039

**Published:** 2016-07-28

**Authors:** Nathan Mih, Elizabeth Brunk, Aarash Bordbar, Bernhard O. Palsson

**Affiliations:** 1 Bioinformatics and Systems Biology Graduate Program, University of California, San Diego, La Jolla, California, United States of America; 2 Department of Bioengineering, University of California, San Diego, La Jolla, California, United States of America; 3 Department of Pediatrics, University of California, San Diego, La Jolla, California, United States of America; Center for Cancer Research, UNITED KINGDOM

## Abstract

Progress in systems medicine brings promise to addressing patient heterogeneity and individualized therapies. Recently, genome-scale models of metabolism have been shown to provide insight into the mechanistic link between drug therapies and systems-level off-target effects while being expanded to explicitly include the three-dimensional structure of proteins. The integration of these molecular-level details, such as the physical, structural, and dynamical properties of proteins, notably expands the computational description of biochemical network-level properties and the possibility of understanding and predicting whole cell phenotypes. In this study, we present a multi-scale modeling framework that describes biological processes which range in scale from atomistic details to an entire metabolic network. Using this approach, we can understand how genetic variation, which impacts the structure and reactivity of a protein, influences both native and drug-induced metabolic states. As a proof-of-concept, we study three enzymes (catechol-O-methyltransferase, glucose-6-phosphate dehydrogenase, and glyceraldehyde-3-phosphate dehydrogenase) and their respective genetic variants which have clinically relevant associations. Using all-atom molecular dynamic simulations enables the sampling of long timescale conformational dynamics of the proteins (and their mutant variants) in complex with their respective native metabolites or drug molecules. We find that changes in a protein’s structure due to a mutation influences protein binding affinity to metabolites and/or drug molecules, and inflicts large-scale changes in metabolism.

## Author Summary

Structural systems pharmacology is an emerging field of computational biology research that aims to merge network and molecular views of biology. Genome-scale models are *in silico*, network models of metabolism, and by integrating the detailed knowledge we can gain from molecular simulations with these models, we can begin to understand whole cell phenotypes at a more complete scale. In this study, we use and integrate a variety of simulation tools at both the network and molecular levels to allow us to understand how a mutation can change an enzyme’s ability to bind to drugs or metabolites. We look at three different enzymes within red blood cell metabolism, and find that these computational tools reflect what we know about them relatively well, and also potentially serve as a workflow for understanding other traits in the overall theme of personalized medicine.

## Introduction

Synergistic advances in pharmacogenomics, genome-wide association studies (GWAS) and next-generation sequencing bring promise to future applications of personalized medicine. Exploring the mechanistic link between human sequence variation and responses to drug therapy is likely to shed light on why certain drugs show a reduced or even harmful effect on specific individuals. For example, if an individual has a specific polymorphism or rare variant, the consequences of administering a given drug are potentially immense if a life-threatening gene-drug association has not yet been identified [1]. While numerous harmful gene-drug associations have been identified from GWAS (and those with significant side effects now have warnings on pharmaceutical labels [2]), screening genome-wide associations across the broad scope of available pharmaceutical compounds is currently limited by both the cost of carrying out such studies [3] as well as a lack of statistical power due to the rarity of deleterious mutations.

To address these limitations, a number of recent studies have developed mechanistic, computational analyses and the construction of omics-based workflows that identify, for example, the mode of action of common drug side effects [4]. Genome-scale modeling enables the analysis of disease-causing mutations in mechanistic detail. Genome-scale models of metabolism (GEMs) encompass the known interactions of diverse biological components, or the reactome of a target organism, into a unified, functional framework. This framework contains all known metabolic reactions, the genes that encode each enzyme, and all metabolites in a given organism and therefore provides a direct mapping from genes, to gene products, to the phenotypic responses of cellular activity. Mapping sequence variations in a gene to changes in the biological states of an entire metabolic network enables characterizing the effects of sequence variation in simplified cellular systems, such as the human erythrocyte [5,6]. Furthermore, a recently updated version of the erythrocyte metabolic model (*i*AB-RBC-283), based on the global reconstruction of the human metabolic network (Recon 2) [7] has been used to study the response of the cell to deleterious single nucleotide polymorphisms (SNPs) as well as drugs with known targets [5,8,9].

Predicting the wide range of possible effects that SNPs and single nucleotide variations (SNVs) can have on structure-function relationships in proteins requires extending a systems-level description to include details from physics-based approaches, such as molecular dynamics simulations. To this end, three-dimensional structures of proteins provide complementary data for further elucidating changes in drug-protein interaction networks. Much attention has been placed on developing bioinformatics tools for the statistical analysis of large-scale data sets, (which contain information on non-synonymous, exonic mutations on individual proteins), and generating hypotheses that explain how mutations affect stability, protein-protein interactions, ligand binding, or catalytic function [10]. Atomistic simulations have been used as a complement to experimental methods to assess changes in relative binding affinities of potential lead compounds to key enzymatic targets [11]. While these approaches are rich in molecular-level details, they are limited in their ability to address *how* significant the observed changes are in the context of an entire biochemical pathway or, ultimately, a whole cell. This limitation thus motivates the need to develop novel workflows that integrate systems-level and molecular-level details to characterize biological processes at graded levels of chemical detail [12–14].

**Fig 1:**
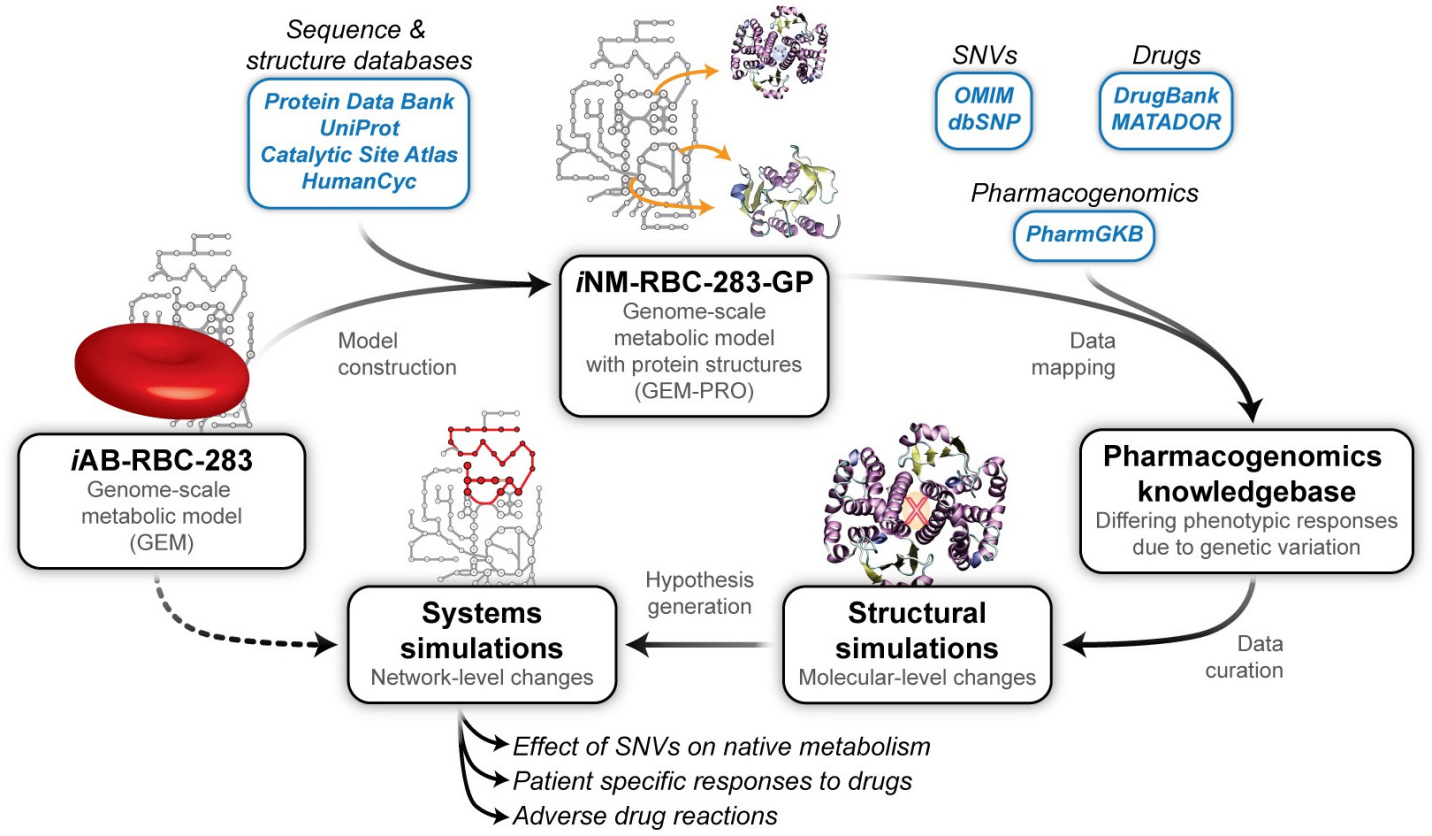
A novel workflow for advancing systems pharmacology. Starting from the genome-scale model of human erythrocyte metabolism (*i*AB-RBC-283 [8]), we integrate information from sequence and structure databases, such as UniProt [40] and the Protein Data Bank (PDB) [30]. Using information from the PDB, experimental protein structures are linked to their respective encoding genes and interacting partners in the metabolic networks. Using homology modeling, representative templates are used to build structural models of target proteins when existing experimental structural information is sparse or missing. The resulting GEnome-scale model of Metabolism with PROtein structures, GEM-PRO, (referred to as *i*NM-RBC-283-GP), presents all of this information in a single database and can be used to generate hypotheses related to cell function in the presence of environmental perturbations. Using other external databases such as the PharmGKB [29], information about known SNPs, drug-related effects, and pharmacogenomic data is used to find promising protein targets that are characterized at the molecular level. Finally, the information gained from structural simulations (e.g. substrate docking and molecular dynamics simulations) can be used as input to guide systems modeling and test hypotheses related to drug-induced effects on metabolism.

The growing field of structural systems biology brings promise to the integration of systems and molecular sciences, enabling applications in personalized medicine [13,15–17], drug discovery [18–20], understanding off target binding [21–23] or mechanisms of action, [24–26] and also to enhance pharmacokinetic/pharmacodynamic models [27]. Here, we build upon previous studies which integrate protein structural information into GEMs [22,23,28], by developing a multi-scale framework to analyze the effects of sequence variation on drug responses in human erythrocyte metabolism (Fig 1). Using genome-scale modeling approaches, we identify key proteins in erythrocyte metabolism that are perturbed in the presence of (i) pharmaceutical drugs and (ii) sequence variants. Using atomistic simulations, we characterize changes in structure and function relationships for different metabolic proteins in the form of drug or metabolite binding differences resulting from reported sequence variants. Finally, we integrate the knowledge gained from these simulations into a detailed genome-scale model of the erythrocyte, allowing for both constraint-based and kinetic methods of analysis to understand the systems-wide effect of these variants.

## Results and Discussion

### Pharmacogenomics in the human erythrocyte

**Fig 2:**
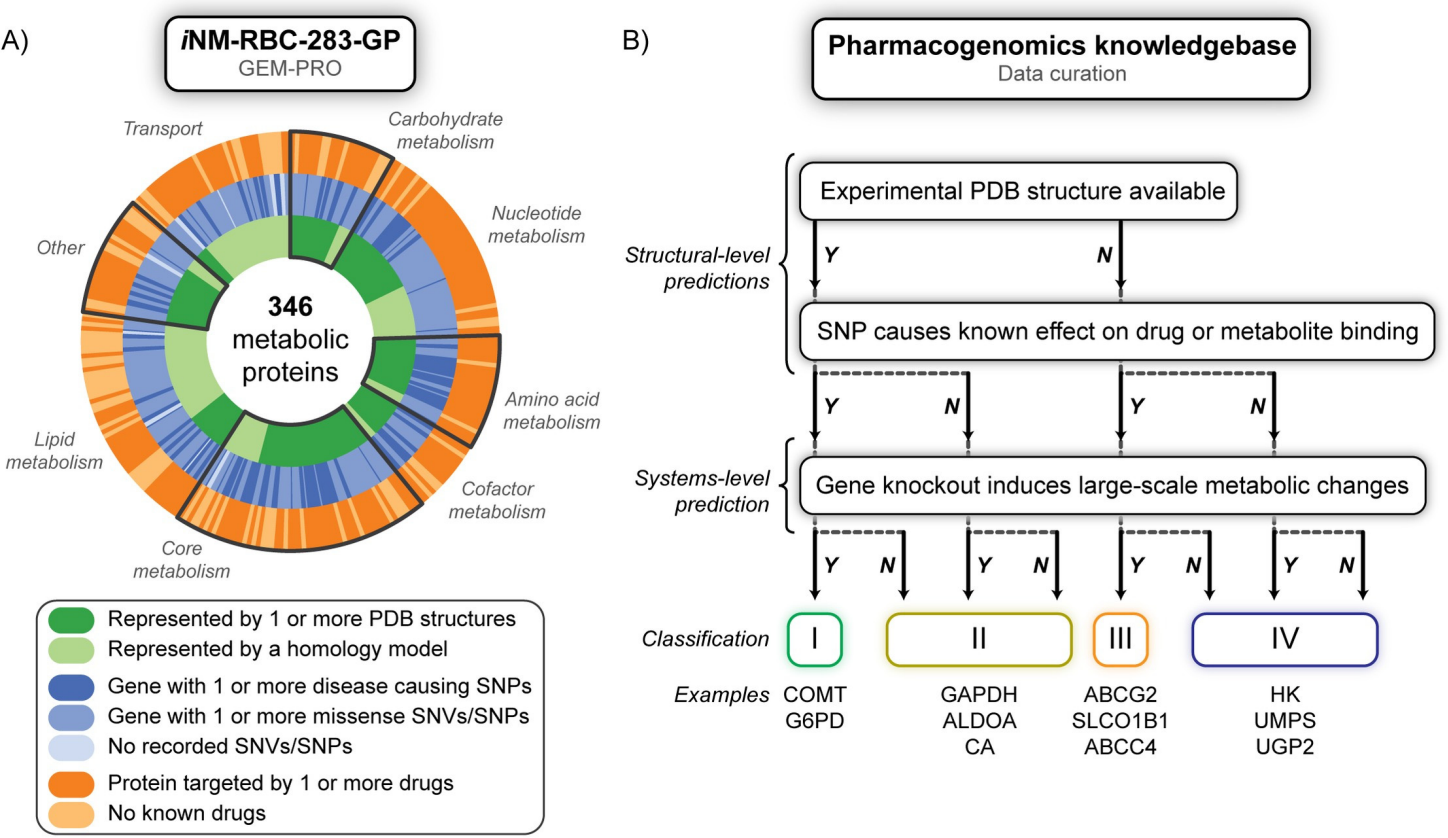
In a), coverage of structural and pharmacogenomics information for the human erythrocyte. The metabolic network is based on 346 proteins, and each narrow slice of the pie chart represents one protein. The innermost circle represents structural coverage by an experimental structure (dark green) or by a homology model (light green). The middle circle indicates if the gene is known to contain at least one disease causing SNP (dark blue), at least one missense SNV or SNP (blue), or no recorded SNVs/SNPs (light blue). The outermost circle includes information from various drug databases, and indicates if that protein is known to be a drug or drug metabolite target (dark orange) or if no drugs target that protein (light orange). Basic subsystems of erythrocyte metabolism are highlighted as regions of the chart. For a full chart of numeric counts for each category and subsystem division see Fig C in S1 Text. In b), pharmacogenomics knowledge base generation. Our knowledge base includes information on: drugs or metabolites that are predicted to bind to/are metabolized by a protein; known associations between a drug and variation within a population; all variation sites that alter the sequence of the protein target. Targets are filtered into four classes based on if there is a protein structure available, if a SNP causes known effects on drug or metabolite catalysis or binding, and finally if the protein itself is important within the context of the import and export of metabolites in the erythrocyte from gene knockout simulations and flux variability analysis (FVA). Included at the bottom are examples of genes that match these classes of information.

We were interested in quantifying the number of proteins in the human erythrocyte metabolism that (i) are known pharmaceutical targets and (ii) have been documented with both disease and non-disease causing mutations (Fig 2(A)). The erythrocyte presents a valuable and tractable model system for studying the effects of human genetic variation on drug metabolism. First, it is widely appreciated that the erythrocyte possesses drug metabolizing capabilities such that extracts of erythrocyte enzymes are commonly used as a general measure of enzyme activity [31,32]. Second, genetic changes that occur in cells other than the erythrocyte are often manifested in the erythrocyte, assuming correct isoforms and similar genetic control [33–36]. The ease of collection of human erythrocyte samples and subsequent purification of enzymes of interest motivates the study of the erythrocyte as an *in silico* model that can be tested against. Lastly, the erythrocyte outnumbers any other cell type in the human body (85% of the total cell count) [37].

Starting from the set of metabolic genes in the genome-scale model, *i*AB-RBC-283 [8], we mapped gene identifiers to cross-referenced information from dbSNP [38], OMIM [39], and UniProt [40]. We find that for 6800 exon coding SNPs in genes which are expressed in the erythrocyte, the majority (>90%) are missense SNPs as opposed to frameshift or insertion/deletion variations. These SNPs map to 247 of the 281 genes (88%) in the erythrocyte model. The majority of these annotated as “disease-causing” map to enzymes within the heme biosynthesis, glycolysis, and galactose metabolism pathways, which is consistent with hemolytic dysfunction. Other non-disease causing SNPs, (or SNPs with unknown associations), occur in nucleotide metabolism. Harmful mutations also tend to alter the type of amino acid much more than non-disease causing SNPs. For instance, mutations from a hydrophobic residue to another hydrophobic residue are quite common, but disease causing SNPs greatly increase this type of amino acid change to a polar, non-polar, or positive amino acid (Fig D in S1 Text).

Our pipeline also identifies variants that potentially influence drug-binding capabilities of respective proteins. Of the metabolic proteins in the erythrocyte, 143 are found to be potential targets for pharmaceutical action. We find 343 drugs (approved, experimental, withdrawn drugs, or drug metabolites) that bind to different proteins in the model [41,42]. In addition, mapping to the PharmGKB database, we find 274 deleterious SNP-drug associations, or documented adverse reactions (i.e., pharmaceutical complications) in patients (referred to herein as SNP-drug association). To summarize, our systems pharmacological database provides details on all documented missense SNPs in erythrocyte metabolism, whether they are causal for disease or cause pharmaceutical complications in a significant percentage of the human population with a sequence variation [29]. In addition, our dataset contains information on drug-binding capabilities of all proteins in the model. This combined source of information for genetic and pharmacological information within the erythrocyte allows for the selection of interesting targets to further analyze with both molecular and systems simulations.

### Mapping protein structures to the metabolic network of the human erythrocyte

To address the structural implications of changes to sequence or drug-binding capacity, we were interested in mapping all protein-encoding genes within the metabolic network of the erythrocyte to their three-dimensional (3D) macromolecular structures. Integration of protein structural data and GEMs has previously been described through the construction of GEnome-scale models of Metabolism with PROtein structures (GEM-PRO). The established pipelines for constructing a GEM-PRO have been recently updated [28]. Applying this procedure for the human erythrocyte metabolic model, we start from the existing GEM, *i*AB-RBC-283 [8], and the final outcome is a mapping of all protein-encoding genes to the 3D structures of their catalyzing enzymes. The selected protein structures have been quality-controlled and ranked to ensure the highest quality structures are retained. The new GEM-PRO model, *i*NM-RBC-283-GP, initially contained structural coverage for 181 of the 346 proteins in the metabolic network (Fig 2(A)), and includes a total of 1766 unique PDB entries (the original GEM is comprised of 281 genes which encode 346 unique proteins). In addition, 312 homology models were obtained for proteins from existing homology model databases [43], using the I-TASSER suite of programs [44].

Our QC/QA pipeline identifies experimental structures and homology models that can be used with high confidence in molecular modeling simulations [28]. Several quality metrics are used to rank-order structures, including: (i) coverage of the wild-type amino acid sequence (with a wild-type being defined as the canonical UniProt sequence); (ii) X-ray structure resolution; (iii) number of missing or unresolved parts of the structure. The final QC/QA statistics indicate that 36% of proteins in the GEM model (125/346) have high quality structural information, whereas the remaining 64% (221/346 proteins) can be represented by template-based and *ab initio* generated homology models (see Fig C in S1 Text for detailed statistics on subsystem coverage).

Interestingly, when we combine the structural data and the pharmacogenomic data, we are able to assess SNP data in the context of protein structural information and derive new association. For example, we find that, on average, disease causing SNPs are 4 Å closer to annotated enzyme active sites than non-disease causing SNPs. All structural annotations, mapped database information, and quality statistics are included as a supplementary database (S1 Database).

### Identifying signature proteins with disease phenotypes

One of the main advantages of assembling a structural systems pharmacological dataset for the erythrocyte is that it can be used to address questions requiring multi-scale perspectives, such as “Can mutating a single amino acid in a protein influence network-level perturbations, and, ultimately lead to disease phenotypes?” Considering the availability of information (pharmacogenomic and structural) that emerged from our mapping efforts, we were interested in focusing on several specific cases that could be studied in greater molecular detail, using a combined systems and molecular modeling approach.

To this end, we assessed the available experimental, pharmacogenomic, protein structural and metabolic information available for all proteins in the erythrocyte model. Given the data collected from publically available datasets (described above), we classified proteins based on: (i) availability of experimental protein structure, drug or metabolite binding information, (ii) known harmful gene-drug associations and (iii) if the knockout of this gene within the context of erythrocyte caused significant changes in metabolite import and export (see Methods), resulting in four different classes of proteins based on these criteria (Fig 2(B)). This categorization mainly aids in the next steps of our contributed workflow, in studying the effects of SNVs on metabolite and drug binding using all-atom molecular simulations.

As shown in Fig 2(B), Class I targets have the most information available, including 3D protein structures (some in complex with a metabolite, drug or analogue), known drug-protein interactions, gene-drug associations, and clinically relevant phenotypic responses to a drug therapy. This group of proteins includes six proteins: catechol-O-methyltransferase (COMT), aldehyde dehydrogenase (ALDH3A1), adenosine deaminase (ADA), glucose-6-phosphate dehydrogenase (G6PD), glutathione peroxidase 1 (GPX1), and uridine 5'-monophosphate synthase (UMPS). Class II targets provide case-studies amenable to experimental testing SNV or drug-induced effects. Class III & IV targets are proteins found to be important in the genome-scale model, but do not have other sources (structural or pharmacogenomic) of information available, and therefore constitute examples of where our molecular modeling framework is useful for filling in missing information (Table B in S1 Database).

**Fig 3:**
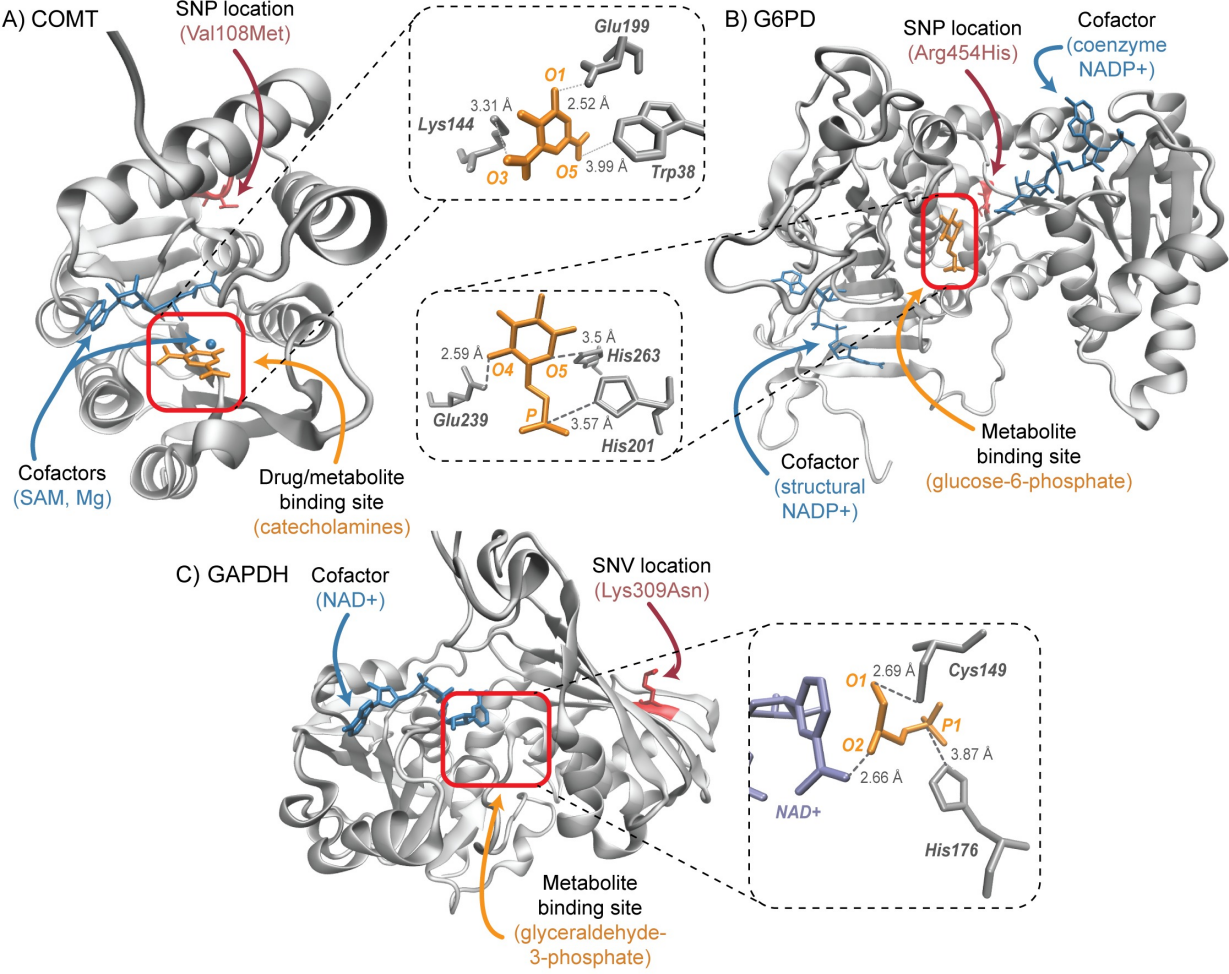
a) Protein structure of COMT (WT) from PDB entry 3BWM. In orange—crystallized position of an inhibitor analog, dinitrocatechol (DNC). In blue, cofactors needed for catalysis, S-adenosyl-methionine (SAM) and magnesium (Mg). In red, the position of the SNP (contained in PDB entry 3BWY). Zoom in—shows the active site of the enzyme with the crystallized DNC bound. b) Protein structure of G6PD (WT) from PDB entry 2BH9. In orange—crystallized position of the metabolite glucose-6-phosphate (G6P). In blue, the cofactor NADP+. In red, the position of the SNP. Zoom in—shows the active site of the enzyme with G6P bound. c) Protein structure of GAPDH (WT) from PDB entry 1U8F. The orange arrow indicates the known binding site of the metabolite glyceraldehyde-3-phosphate (G3P), which was not crystallized in the experimental structure. In blue, the cofactor NAD+. In red, the position of the SNV. Zoom in—binding site interactions of G3P in *E*. *coli* PDB entry 1DC4.

Here, we focus the rest of this study on three distinctive proteins in erythrocyte metabolism (Fig 3): (i) catechol-O-methyltransferase (COMT), a class I protein (according to our above classification scheme); (ii) glucose-6-phosphate dehydrogenase (G6PD), a class I protein; (iii) glyceraldehyde-3-phosphate dehydrogenase (GAPDH), a class II protein. For the purpose of validation, we study the class I proteins, which have ample experimental, structural and pharmacological data associated with their roles in metabolism. To assess the predictive value of this workflow, we study the class II protein, a rare variant where population data was not available to understand the impact of documented sequence variants. Such an example serves as a demonstration for how this structural systems biology framework can be used in the absence of experimental and pharmacological data. The targets chosen for this study and their pharmacogenomic importance are outlined in Table 1.

**Table 1:** Signature proteins that impact erythrocyte metabolism and drug-induced phenotypes.

Gene	Protein name	Native metabolite	Cofactors	WT PDB	Mutant PDB	SNP/SNV	Drugs known to bind	Number of exonic SNPs
COMT	Catechol-O-methyltransferase	Dopamine, epinephrine, norepinephrine	SAM, Mg	3BWM, 3A7E	3BWY	Val108Met	Tolcapone, entacapone,	113
G6PD	Glucose-6-phosphate dehydrogenase	Glucose 6-phosphate	NADP+	2BH9, 2BHL	Numerous	Arg454His	Phenobarbital metabolites	206
GAPDH	Glyceraldehyde-3-phosphate dehydrogenase	Glyceraldehyde 3-phosphate	NAD+	1U8F	Modeled	Lys309Asn	N/A	82

### Molecular effects of sequence variation in protein-drug interactions

**Fig 4:**
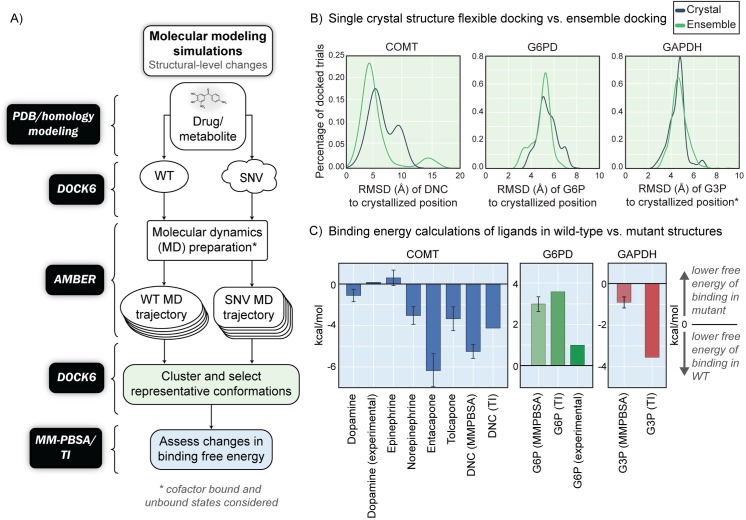
a) Molecular modeling frameworks used for molecular simulations of metabolite and drug binding differences between wild-type and mutant (SNV/SNP) proteins. In the first step, docking is first carried out on experimental or modeled protein structures. From molecular dynamics simulations, an ensemble of structures is generated from the long-time sampling of conformations that cannot be studied from a single, static structure (e.g. crystallographic structure). These ensemble structures provide multiple thermodynamic states of the protein that enable docking and analysis of binding free energy estimates. The overall goal of using these molecular modeling frameworks is to quantify the relative differences in the binding affinity of metabolites and drugs to wild-type and mutant proteins. Once these differences are computed, the ratios will be used to guide systems-level simulations. b) RMSD of predicted ligand poses of DNC to the original crystallized position based on docking trials to only the crystal structure (blue) versus utilizing an ensemble of structures (green). c) Differences in binding free energies from MM-PBSA calculations in wild-type vs. mutant proteins. A negative value indicates a lower predicted binding free energy to the wild-type protein, which corresponds to a higher binding affinity.

The next stage of our proposed workflow builds on previous methods [22,23,45,46] and leverages systems modeling with molecular dynamics (MD) simulations. How SNPs/SNVs affect structure/function relationship is a question that requires analysis beyond a comparison of crystal structures. Here, we take advantage of using an ensemble of protein conformations, generated from explicit solvent MD simulations, to study the effects of clinically relevant SNVs/SNPs on drug and/or native metabolite binding (Fig 4(A)).

#### Catechol-O-methyltransferase (COMT)

The activity of COMT in the erythrocyte, along with the inheritance of specific polymorphisms, is often used as a biomarker for different diseases, such as Parkinson’s disease or schizophrenia [47–49]. COMT plays a critical role in the degradation of catecholamines, a class of chemicals that mostly function as neurotransmitters in the human body [50], making it a prime target for further elucidating the effects of this SNP on protein-drug interactions. Further, COMT plays a key role in the erythrocyte, and its relationship to pharmacogenomic implications is likely to be applicable in other systems in the human body [51]. Of particular interest is the missense mutation, Val108Met, (i.e. Val158Met in the membrane bound version; dbSNP ID rs4680), which may cause changes in the protein’s response to drug inhibitors [52]. While the crystallographic structures for both wild-type and SNP variants are available, minimal structural changes between the protein backbone of these two proteins are observed (i.e. they align with a 0.2 Å root mean squared deviation, RMSD (Fig E in S1 Text)) [53].

We were interested in characterizing the binding mechanism of COMT, when it is in complex with either its native substrates (i.e. dopamine, epinephrine, norepinephrine) or known inhibitors/inhibitor analogs (e.g. dinitrocatechol, tolcapone, entacapone). Flexible molecular docking of dinitrocatechol (DNC), which is co-crystallized in both PDB structures, to the crystal structures of both wild-type and SNP variants gave a RMSD of less than 1 ‎Å (of the drug backbone with respect to the original co-crystallized position) (Fig F in S1 Text). We find that docking without the presence of the cofactors, (i.e., S-adenosyl methionine and a magnesium ion), slightly increases the RMSD (by 1.5 Å) of the binding pose, as expected due to the stabilizing features and steric constraints of these cofactors [54,55]. Similar to DNC, docking of the native metabolites to the crystallographic structures retrieved binding poses within a RMSD of 2 Å of the original bound position (comparing equivalent atoms of the co-crystallized inhibitor DNC) (Fig G in S1 Text). The best docked poses of the two drug molecules (tolcapone and entacapone) were initially reported about 10 Å away from the known binding site, which motivated ensemble docking of both wild-type and variant proteins to understand the conformational space which was not represented in the crystal structure.

To generate an ensemble of conformations, we performed MD on both the wild-type and SNP variant proteins, in complex with their cofactors. We find that docking DNC to an ensemble of representative structures provides an increased accuracy in the final binding pose (Fig 4(B), COMT panel), compared to docked poses to only the single crystallographic structure, consistent with previous studies [56–60]. Ensemble docking of the catechol-like drugs and metabolites retrieved binding poses to within 5 Å of the original crystallized position in 72% of clustered snapshots from an MD trajectory. Furthermore, ensemble docking to wild-type COMT has a higher frequency of reproducing the crystallized binding orientation compared to the SNP variant (Fig H in S1 Text). Following clustering of promising binding poses, we performed molecular dynamics for each of the proteins in the ligand-bound states and computed the free energy difference between the wild-type and variant proteins using MM-PBSA [61]. As shown in Fig 4(C), we find that for the majority of cases, the mutation V108M leads to a decreased affinity (i.e. an increase in relative binding free energy between wild-type and mutant protein, resulting in a negative ΔΔG (ΔG_WT_—ΔG_SNP_)) to a majority of the native metabolites and drug molecules (excluding epinephrine; see Table K in S1 Text). These findings are consistent with experiments that find the SNP variant to be less stable and less active, along with variant human subjects that respond less to drug therapy [52,62,63], yet no significant experimental differences were found with dopamine binding to the mutant [62].

#### Glucose-6-phosphate dehydrogenase (G6PD)

G6PD catalyzes the oxidation of glucose-6-phosphate (G6P) to 6-phospho-gluconolactone (6PG) within the pentose phosphate pathway, while maintaining the global concentration of NADPH in the erythrocyte [64], required for protecting the cell from oxidative damage. There are more than four hundred sequence variants [65], of which many are implicated in hemolytic anemia and can be heavily influenced by drug side effects or a compromised immune system [66]. One particular missense mutation, referred to as the “Andalus” SNP (Arg454His; dbSNP ID rs137852324), has been classified as causing chronic nonspherocytic hemolytic anemia [67]. Structurally, the mutation occurs 21 Å from the substrate binding site (Fig 3(B)) and is expected to impact a salt bridging interaction with Asp286, potentially destabilizing the local structure of the protein. Notably, this arginine residue is highly conserved throughout organisms, reinforcing its structural and functional importance [68].

Similar to COMT, docking trials were carried out on wild-type G6PD and SNP variant structures. The wild-type structure was modified to generate the SNP variant and structural changes resulting from the sequence change were monitored during a 100 nanosecond trajectory. As expected, the salt bridging interaction between the mutated residue and Asp286 was eliminated (Fig I in S1 Text). We performed ensemble docking simulations of various substrates to representative structures from the MD trajectory and found that, in 95% of the docking trials, G6P binds within 5 Å of the known active site of G6PD (Fig 4(B)). Although we do not observe large-scale differences in the docking poses of G6P in wild-type versus SNP variant proteins (Fig J in S1 Text), binding free energy calculations indicate that the SNP variant has an increased binding affinity to the native substrate: G6P binds to the SNP variant with a ΔΔG = 3.00 ± 0.68 kcal/mol (ΔG_WT_—ΔG_SNP_). We find that this value is consistent when comparing to higher accuracy methods (e.g., from thermodynamic integration (TI), we find ΔΔG = 3.59 kcal/mol) (Fig 4(C), G6PD panel). Experiments demonstrate that this mutation markedly increases the binding affinity of the native metabolite G6P in the variant while radically decreasing the overall turnover rate ($K_{m}^{WT}$ = 52 ± 4 μM, $K_{m}^{SNP}$ = 9.71 ± 0.67 μM, calculated ΔΔG = 0.99 kcal/mol) [67]. Additionally, we find the SNP variant to have an increased binding affinity to the product of the reaction, 6PG (ΔΔG = 5.81 ± 3.11 kcal/mol), and a drastically decreased binding affinity to the cofactor NADP^+^ (ΔΔG = -13.057 ± 2.58 kcal/mol) at the secondary “structural” cofactor binding location [66,69]. These may also be factors that contribute to the decreased turnover rate, such as due to a slower product release compared to wild-type behavior or enzyme instabilities caused due to a lower population of bound NADP^+^.

#### Glyceraldehyde-3-phosphate dehydrogenase (GAPDH)

GAPDH is an enzyme within the glycolytic pathway that catalyzes the conversion of glyceraldehyde 3-phosphate (G3P) to glycerate 1,3-bisphosphate, utilizing the cofactor NAD^+^. It operates as a homotetramer, and a conserved cysteine residue (Cys149) is essential for its catalytic function [70]. Designated in this study as a Class II pharmacogenomic enzyme, it does not have any recent documented variants with phenotypic data, but from HapMap population sequencing data, a missense mutation, Lys309Asn (dbSNP ID rs11549334) was identified and predicted (using PolyPhen2 and SIFT) to be deleterious and/or disruptive (Table J in S1 Text) [71,72]. This mutant is found to occur 19 Å away from the binding site (Fig 3(C)). As with much of the sequence of GAPDH, this residue is conserved throughout eukaryotic organisms [73], and thus observed changes are rare and likely marked as deleterious according to these predictive algorithms.

The Lys309Asn mutant structure was generated by modifying the sequence of the experimental wild-type structure and monitoring structural changes during a 100 nanosecond trajectory. In this case, ensemble docking resulted in a slight trend for more correct binding poses in the WT ensembles when compared to the mutant (Fig 4(B), Fig K in S1 Text). Clustering of the docked poses was carried out based on ligand-protein interactions obtained from literature (Table I in S1 Text) [74–76]. Computing the free energy binding difference for G3P between wild-type GAPDH and mutant protein, we confirm that the wild-type binding affinity is stronger than that of the mutant variant (ΔΔG = -3.55 ± 0.6 kcal/mol) (Fig 4(C), GAPDH panel). The binding of the cofactor (NAD^+^) was found to have similar binding affinities in both forms of the enzyme (ΔΔG = -0.5504 ± 1.8 kcal/mol). Due to the highly conserved nature of this specific residue and the suggestion of a decreased binding affinity to the native metabolite G3P, our predictions are consistent in that it may be a cause of enzymopathy.

### Systems-level effects of sequence variation related to drug responses

**Fig 5:**
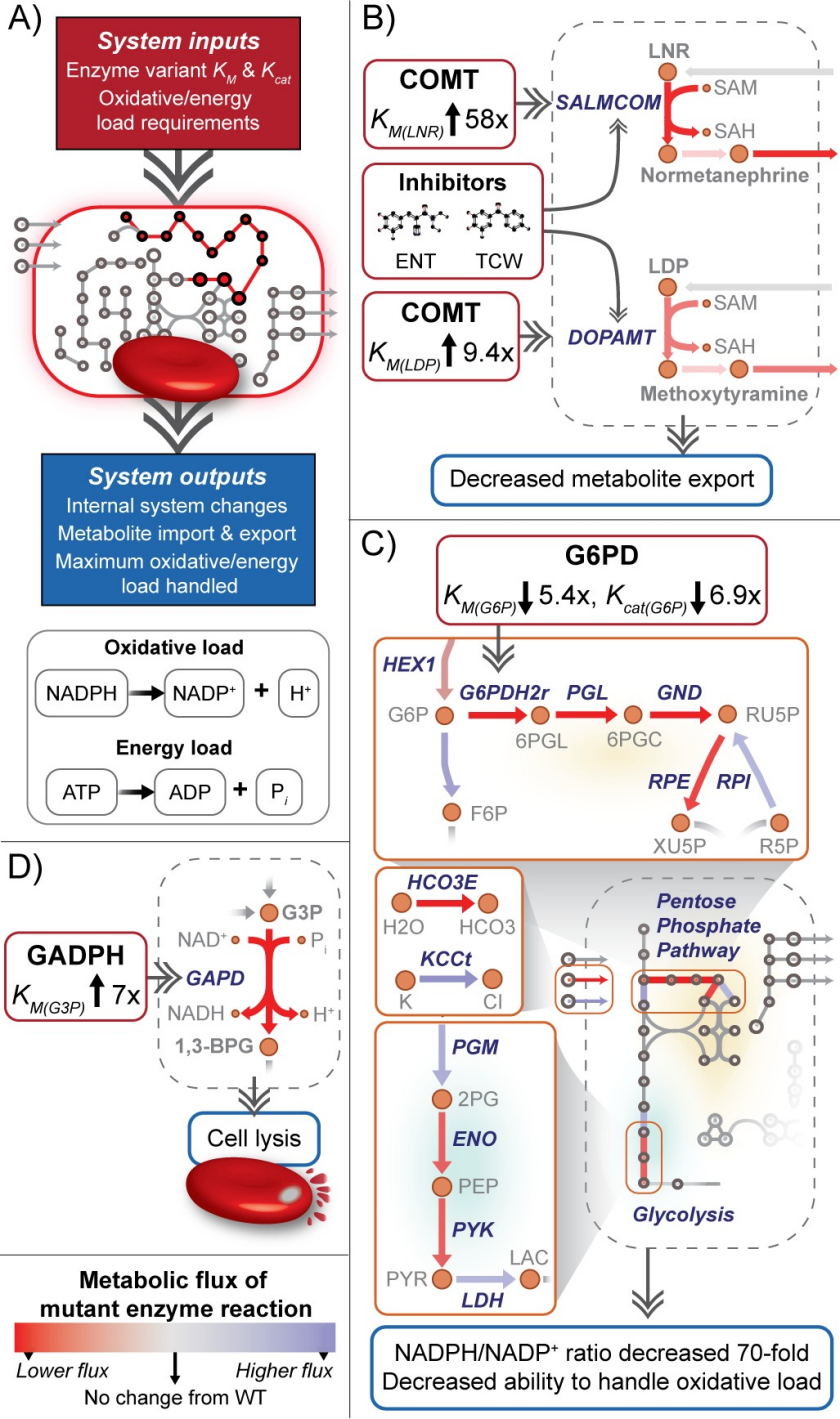
a) Systems modeling framework used in this study. Inputs used for constraint-based and kinetic modeling are derived from molecular modeling calculations and experimental data when available. In order to understand how small-scale changes from enzyme variants affect the entire system, we look at the internal system changes (in reaction flux and metabolite concentration), differences in metabolite import & export, and how the cell handles an increase in oxidative or energy loads. Oxidative load is defined as the conversion of NADPH to NADP^+^, whose rate of reaction is increased under states of oxidative stress. Energy load is defined as the use of ATP. For all panels, the change in metabolic flux is colored by a difference from the wild-type flux state, red being a decreased flux in the mutant state and blue being an increased flux. b) Constraint-based modeling for the mutant COMT enzyme. The SNP is predicted to decrease the binding affinity of the enzyme in norepinephrine and dopamine metabolism. Increasing the K_m_ (predicted) of COMT for the respective reactions leads to decreased flux and as a result decreased export of their methylated counterparts. Inhibitors tolcapone (TCW) and entacapone (ENT) are also predicted to have a lowered binding affinity to COMT, leading to similar effects. c) Kinetic modeling for the mutant G6PD enzyme. Decreases of the K_m_ (predicted and experimental) and of the K_cat_ (experimental) lead to major systemic changes of the pentose phosphate pathway and glycolysis. The ratio of NADPH to NADP^+^ greatly decreases and subsequently the oxidative load able to be handled also decreases. d) Kinetic modeling for the mutant GAPDH enzyme. The cell is unable to handle the predicted increase in K_m_ (predicted) and results in an infeasible state of the model, corresponding to cell lysis.

While understanding protein-drug interactions provides information on *how* sequence variation changes protein structure and reactivity, evaluating the downstream effects of these changes requires a systems-level perspective (Fig 5(A)). Changes in metabolic networks can be assessed using a variety of systems methods including constraint-based and kinetic modeling techniques [5,77–79]. To test the susceptibility of the metabolic network of the human erythrocyte to the harmful variants detailed above, we utilized both constraint-based modeling of the *i*AB-RBC-283 model [8] and a recently developed *in silico* kinetic rate law model derived from the Mass Action Stoichiometric Simulation (MASS) approach [80,81]. For a number of proteins, disease causing mutations can cause systemic changes within the metabolic network or in the transport of certain metabolites [8,82]. With regards to the erythrocyte, understanding these differences in metabolite transport can be correlated with changes in metabolite concentrations within biofluids, which potentially expands the use of this model as a diagnostic tool for human disease. Similar perturbations can also be linked to the specific phenotypic responses of the erythrocyte, such as to drug treatments, or the ability to respond to changes in oxidative (rate of NADPH use in order to combat oxidants) or energy (rate of ATP use) load [5].

#### Catechol-O-methyltransferase (COMT)

As COMT is not present in the core erythrocyte kinetic model [80], we therefore turned to constraint-based modeling techniques, utilizing the entire genome-scale model of the erythrocyte. We used the established Markov Chain Monte Carlo-based (MCMC) sampling approach [83] to calculate distributions of all feasible flux states for both wild-type and SNP systems. Ligand binding differences between wild-type and SNP variant (computed from the molecular simulations) were integrated into the erythrocyte model by altering the reaction flux bounds, which represent the rates that metabolite flow through a reaction. We used the relative ratio of the binding affinity differences to effectively constrain the quantitative relationship between the wild-type and mutant metabolic state as well as the difference in behavior of the enzyme under a drug load.

Our findings suggest significant changes in the uptake of dopamine and norepinephrine, and the secretion of their methylated counterparts (Fig 5(B)) as a result of the sequence variant. In contrast, for epinephrine, the computed binding free energy difference in wild-type and SNP protein was positive (i.e. it binds more strongly to the mutant), which did not influence network analysis of the uptake or secretion of its associated metabolites. Furthermore, the mutant COMT decreases the effectiveness of the drugs entacapone and tolcapone, which is again reflected by an increase in the secretion of the methylated metabolites when compared to the wild-type cell inhibited by these drugs. These findings are consistent with previous studies related to entacapone, which report decreased efficacy of entacapone in individuals with the SNP (Met108) [52], though it may be dependent on different human-specific characteristics [84] and tolcapone, which has a reported increased efficacy in individuals with the wild-type (Val108) [85]. These findings oppose the previous claim that the genotype did not contribute to the clinical response [86].

#### Glucose-6-phosphate dehydrogenase (G6PD)

In patients with G6PD deficiency, the most common symptom is hemolytic anemia resulting from the erythrocyte’s loss of ability to respond to oxidative stress. This ability can be measured by simulating an increase in the oxidative load within a kinetic modeling framework. The predicted increase in binding affinity of G6P to the mutant enzyme corresponds to the experimentally calculated binding affinity reported in [67]. If we assume the same catalytic rate (*K*_*cat*_) of the reaction carried out by this enzyme, the cell’s ability to respond to an oxidative load does not decline. Though the ratio of NADPH to NADP^+^ increases, it does not lead to a significant increase in the oxidative load tolerated when compared to the baseline, wild-type model. Integrating the experimentally measured *K*_*cat*_ from [67], however, drastically reduces the ratio of NADPH to NADP^+^ and subsequently lowers the maximum tolerable oxidative load of the cell under stress conditions (Fig 5(C)). Incorporating these kinetic parameters within the erythrocyte model, we find specific systemic effects that correspond to the classification of this SNP as a “severe deficiency with intermittent hemolysis” by the WHO [87]. This behavior is also observed in constraint-based modeling after decreasing the flux through the corresponding reaction and induces significant changes (defined as <40% the original, wild-type flux span, see Methods) in the glutathione reductase pathway, which utilizes NADPH to combat oxidative stress.

#### Glyceraldehyde-3-phosphate dehydrogenase (GAPDH)

GAPDH deficiencies in humans are rare, and mostly cause mild hemolytic anemia [88]. By integrating the relative change in *K*_*m*_ of the mutant, based on binding free energy computations, into the erythrocyte kinetic model, we observed that this change led to lethality (Fig 5(D)). Smaller relative changes in the *K*_*m*_ or *K*_*cat*_ (compared to wild-type) were not lethal and did not impact the ability of the erythrocyte to respond to an increase of oxidative or energy load, suggesting that only a small degree of change in protein structure and reactivity may be tolerated. This finding is consistent with studies in mice where those with lower activity mutants or those heterozygous for a lethal mutant rarely showed symptoms, while those homozygous for a mutant encountered mortality at the development stage [89]. The human subject with this annotated variant (within the HapMap dataset) was noted as having a heterozygous form of this specific mutant, which would explain the non-lethality observed. It is important to note that GAPDH is involved in several non-metabolic processes [90], and while variation of the enzyme sequence may be tolerated to a certain extent, these additional processes may be impacted due to the causal effect of this mutation.

### Conclusion

Here, we propose a framework for mapping protein structural information to genome-scale models of human erythrocyte metabolism for the characterization SNP-drug associations. Three case studies presented in this contribution point to the complexity of pharmacogenomic associations and being able to conduct integrated *in silico* simulations that extend from the molecular scale to the systems level. Using parameters from molecular simulations to guide genome-scale modeling, we are able to study how changes in protein structure and binding affinity influence the phenotypic states of an entire metabolic network. We find that the union of genome-scale modeling and molecular, physics-based methods, presents, to the best of our knowledge, the first workflow capable of systematically integrating data from pharmacogenomics research, in conjunction with 3D high resolution protein structural information, to model changes on both the pathway (i.e. metabolic network) and molecular (i.e. protein) scales. The information gained through molecular modeling simulations can be utilized to supply parameters to both kinetic models and constraint-based modeling approaches and has been found to be amenable to the study of other enzymopathies [5,91]. Our findings indicate that there is consistency between experimental and computational trends in substrate and drug compound binding in wild-type versus mutant proteins.

Currently, most systems biology approaches lack the ability to utilize insights from structure-based analyses related to metabolite and/or drug binding. Fortunately, atomistic molecular simulations have evolved to become powerful tools for the characterization of binding mechanisms and as such constitute valuable assets for systems modeling. Extending analysis beyond crystallographic structures through the use of ensemble confirmations substantially enhances the predictive scope of docking methods by identifying alternative binding modes for a drug molecule [56–60]. Ensembles of the thermodynamically accessible states of a protein, generated from molecular dynamics, allows for the mechanistic characterization of how sequence and structural variation may influence metabolite or drug binding [92].

The scalability of this workflow is mainly limited (i) to the documentation and experimental analysis of exonic SNVs/SNPs, and (ii) by the execution of molecular dynamics simulations, which takes a significant manual effort and requires high performance computing resources. For the second point, certain efforts have already shown that high-throughput simulations using classical MD can be performed on large numbers of proteins [93,94]. However, performing high accuracy computations on a systems scale is currently intractable, due to the intense computational and time requirements of quantum-based simulations or free energy calculations. Therefore, a trade-off between accuracy and cost must be considered (see Fig B in S1 Text and recent reviews on the subject [95–97]). In light of these limitations, we find that the additional information gained from protein structure greatly contribute to our understanding of causal mutations and can assist in selecting protein targets for more detailed molecular studies. Thus, when combined with other developing frameworks [4] and experiments [98], the contributed workflow provides a first step in the translation of Big Data in the pharmaceutical industry to practical therapeutic applications and is expected to have a positive transformative impact on the fields of systems medicine, population studies and drug discovery efforts.

## Methods

### GEM-PRO construction

The techniques used here are a consolidation of 4 previous methods to add protein structural information to genome-scale models [22,23,99,100], and described in detail in [28]. To do so, the SBML model of the erythrocyte genome-scale model was first obtained from the BiGG Models website (http://bigg.ucsd.edu/models/iAB_RBC_283) [101], and all gene IDs were mapped to their corresponding amino acid sequences (UniProt and RefSeq entries). This model differed from the construction of previous GEM-PROs due to the appearance of protein isoforms, and required additional manual mapping to ensure correctness. Gene isoforms led to inconsistencies between database entries and additional difficulty linking to available homology models (discussed in the section “Homology Modeling”). Additional QC/QA steps were taken in order to ensure the correct sequence was being retrieved, as described below.

#### Mapping to UniProt accession numbers

For a given gene in *i*AB-RBC-283, there are a number of associated isoforms, annotated as the gene name and a isoform number, separated by a decimal (eg. "Aldoa.1"). We take the gene name, which is taken from the corresponding gene in Recon 2, obtain the Entrez gene ID [102], and directly map this to its corresponding UniProt accession code (UAC). Then, we directly map isoform numbers to available isoforms in the UAC entry (Fig A in S1 Text, top panel). These are annotated with reviewed isoform-specific sequences, allowing us to filter for the correct experimental PDB structure in later stages.

#### Mapping to RefSeq and Ensembl identifiers

In some cases, the number of isoform sequences annotated in *i*AB-RBC-283 does not match the number of isoforms available in UniProt. For these, we generated a separate mapping pipeline to the RefSeq and Ensembl databases [103]. The Bioservices Python package [104] and Ensembl Biomart tables [105] were used in order to first map the gene IDs without their isoform identifier to their corresponding entries, and then back to isoform IDs according to the transcript name as listed in Ensembl (see Fig A in S1 Text, bottom panel). The information here was also utilized in order to cross-reference what was successfully mapped with the UniProt mapping service. Once the correct isoform entry was found, available PDB mappings were found using the entry ID (RefSeq or Ensembl Protein), or by sequence alignment to the PDB. We note that the difficulty in mapping isoforms and inconsistencies between databases points to a larger need of consistency and standardization for this biological property.

#### Homology modeling

We have filled in the gaps where there are no experimental structures by querying previously generated databases of I-TASSER homology models for *H*. *sapiens* [43], and manually generating homology models for genes that were not part of these databases [44]. The I-TASSER Suite version 4.4 was utilized for the construction of missing structures, and provides an especially useful method in modeling splice isoforms, which are specialized in the erythrocyte [106]. In the final GEM-PRO data frame, we note where available homology models have been mapped to their respective genes. We also include additional information in the data frame that explains the type of computational prediction method used to model the protein structure (e.g. template-based versus ab initio), the corresponding URL (for downloading the homology file from the source), the label (i.e. the identifier of the model given by the homology model database), and information related to the confidence of the homology model (e.g. C-score), the native (homologous) template used for the model, etc. All columns added to the master data frame from this stage are preceded by a ‘i’ for I-TASSER. It is important to note that certain PDB structures with unresolved residues or gaps in the structure can also be homology modeled to enhance the structural coverage of the amino acid sequence. Quality scores for each model are included as PSQS and PROCHECK scores [107,108].

#### QC/QA procedure

For the purpose of molecular modeling, it is important to select high-quality starting structures when conducting docking or molecular dynamics simulations. On a genome-scale, an automatic ranking system becomes a requirement if there are multiple structures or homology models that represent one gene. The main objective of this section is to discuss the quality assessment and quality control of the data that has been thus far mapped to the metabolic network reconstruction. In previous versions of GEM-PROs, experimental structures were additionally classified and ranked according to whether a protein was bound to a native metabolite or ligand, in order to ensure proper binding predictions. While the updated version of the GEM-PRO modeling framework does not include the bound state of a protein as a target characteristic in the quality control pipeline, this data is accessible in the knowledge base. Instead, we are mainly interested in quantifying the general quality attributes of the experimental structure of the protein.

An ideal starting platform for higher-level modeling methods are experimental protein structures without missing residues (especially at the interior of the protein) and 100% sequence identity compared to wild-type. To determine which experimental structures required further modeling or modification, we devised a scoring metric that ranks each PDB based on 1) the coverage of the wild-type amino acid sequence, 2) the resolution, and 3) the similarity of secondary structural features between the PDB structure and its corresponding homology model. The final outcome of the quality assessment is the classification of experimental structures into three groups: (i) high quality structures requiring no modification; (ii) high quality structures requiring minimal (site-directed) modification and (iii) low quality structures requiring homology modeling. For more information on the ranking scheme, please see S1 Text and [28]. All protein structure files following ranking and quality control are included within S3 Database.

### Genetic variation, drug-target interactions, and essential genes

Previous work was done to map data from the Online Mendelian Inheritance in Man (OMIM) database in order to find disease causing mutations that could map to erythrocyte proteins [8]. We also collected all known SNPs from dbSNP, and filtered them down to variations in exons that could be studied utilizing protein structure information. Information was additionally cross-referenced with UniProt variant annotations [109].

There are a number of drug target databases that were queried for this study. DrugBank was used in a previous study to gather drug targets based on sequence [8]. In order to be as comprehensive as possible, we also obtained data from ChEMBL [110] and MATADOR [42], with MATADOR providing annotations for indirect interactions. With this, we were able to verify targets that appeared in all 3 databases. Drug adverse effects due to variation were mainly gathered from the PharmGKB, a pharmacogenomics database with information from clinical studies, research articles, and individual cases [111]. The PharmGKB further annotates for the significance of an association, as well as details of the clinical trial or GWAS study carried out. Finally, the DrugBank contains a simple list of SNP-drug associations in their SNP-ADR and SNP-FX sub-databases [41], which was cross-referenced with all information found in the PharmGKB.

As a final source of parameters for validation of our model, experimentally determined kinetic values for binding of a drug or inhibitor to a target (wild-type as well as mutant) were obtained from BRENDA and the BindingDB [112,113]. As expected, information for this step was much sparser than the previous information, which indicates the need for experimental assays if we are to validate the predictions made from this model. For the targets in this study, we also manually searched for additional information from published biochemical studies.

Finally, for the selection of interesting targets to study with molecular and systems modeling techniques, we also wanted to understand the essentiality of each gene within the erythrocyte model. Gene knockouts were performed for each gene contained within *i*AB-RBC-283, as per [8]. A gene was marked as interesting to study within the context of the erythrocyte if there were significant changes within the reaction fluxes of metabolite import and export through the membrane using flux variability analysis (FVA) simulations [114]. In order to detect these significant differences, all reaction fluxes were compared to the normal “wild-type” state of the cell. Specifically, similar procedures to Shlomi et al. and Bordbar et al. were followed [8,82]. Changes in exchange fluxes were categorized into i) activation/inactivation, ii) shift to a fixed direction, iii) a change in magnitude of flux, or iv) no change (refer to [8], Fig 5). For changes in magnitude of flux, if the new flux span (defined as maximum flux—minimum flux) was less than 40% of the original flux span, it was considered to be a significant change.

### Molecular modeling and docking

Experimental PDB structures or homology models representing the genes of interest in this study were taken from the GEM-PRO data frame following ranking and QC/QA. Mutant forms of the enzymes were either taken directly from the PDB, if available, or modeled by point mutations of the structure. Next, the general approach for each target was to first understand the binding position and energetics of either the native metabolite or a drug of interest to a wild-type protein structure and its corresponding mutant. Flexible docking simulations using DOCK6 were carried out with default parameters and binding sites defined when known [115]. Furthermore, simulations were conducted with and without cofactors, to account for competitive binding drugs or cases where the order of substrate binding was not known. To compare flexible docking results to ensemble docking, simulations were repeated under different random seeds for a total of 500 docking runs.

### Molecular dynamics simulations and ensemble docking

Molecular dynamics simulations were run utilizing the PMEMD module of the AMBER14 toolkit [116]. Initial parameterization of ligands and cofactors were carried out utilizing the Gaussian 09 software [117] or obtained from previously published data sets (see S1 Text for protein-specific methods and S2 Database for parameter sets). For generating topologies as input to AMBER, 99SB force field charges and atom types were then used and then solvated in a periodically repeated TIP3P 12 Å water box with counterions being added as needed (Na^+^ or Cl^-^). Minimization was carried out under constant volume conditions at while being heated to 300 K. Structures were then equilibrated under constant temperature and pressure conditions with restraints being released. Finally, the structures were run in production phase of 75 ns or more under a Langevin thermostat and Particle Mesh Ewald (PME) cutoff of 12 Å.

At least 4 separate MD simulations (representing WT and SNP structures in cofactor unbound and bound states, more for additional cofactor bound states) were carried out on each enzyme (see Tables D-F in S1 Text for all simulation information). Every 100 frames from these trajectories were utilized as input for ensemble docking of the substrate of interest.

All docked positions were clustered into 5 representative poses based on the distances from known binding residues. Specifically, distances from 3 known binding or interacting residues to the atoms of the drug or metabolite were calculated for each extracted frame, and k-means clustering of the Euclidean distance separated these frames into 5 distinct binding modes for use in further simulation. These docked positions were subject to additional MD production runs of 10 ns each, in order to examine the stability of the bound position and if they would converge into one distinct pose. We conducted free energy calculations for each of the ligands in the cofactor bound state of the WT and SNP enzymes. MM-PBSA calculations were carried out to predict the difference in free energies of binding (ΔΔG). The binding energies of all 5 representative conformations were averaged per ligand, and the resulting value indicates if the ligand is more favorable to bind to WT (negative ΔΔG) or SNP (positive ΔΔG) structures.

### Binding energy calculations

MM-GBSA/MM-PBSA calculations utilizing the MMPBSA.py script available in the AMBER14 toolkit were carried out on the 10 ns simulated receptor-ligand complexes [61]. The first nanosecond of simulations was discarded before running calculations to account for initial stabilization of the docked ligand. Thermodynamic integration (TI) calculations were calculated utilizing the Simulated Annealing with NMR-derived Energy Restraints (SANDER) module within AMBER14 [118]. The dual topology paradigm was utilized with a three step alchemical transformation, with state 0 representing a wild-type enzyme and state 1 the mutant form. Step 1 carried out the decharging of the WT utilizing 10 λ points and simulations of 1 ns each. Step 2 transformed the residue atoms of the WT to the SNP again utilizing 10 λ points and simulations of 1 ns each. Step 3 carried out the recharging of the mutant residue atoms with the same number of λ points and simulation time. This was run for both ligand bound and unbound states. Finally, the change in potential energy of the system with ligand bound was calculated by integration over the λ points and subtracted from the ligand unbound state. For full information on docking, MD, MM-PBSA, and TI parameters, please refer to the section entitled “Molecular modeling simulations” in S1 Text.

### Systems modeling

The constraint-based modeling approach was carried out for all enzymes in this study by simulating a normal (wild-type) and perturbed (mutant) erythrocyte condition utilizing FVA followed by a Markov chain Monte Carlo (MCMC) based sampling approach [83,91,119]. Previous simulations for identifying biomarkers have simulated perturbed states by setting the upper and lower bounds of flux through affected enzymes of the cell to 0, effectively mirroring a full gene inhibition, and then analyzing the exchange conditions [8,82]. For the purposes of this study, we are now able to understand the relative differences in native metabolite catalysis utilizing the ratio of differences in the binding affinity between wild-type and mutant forms of the enzymes. This ratio was then converted into a ratio of flux in wild-type to mutant enzymes, assuming equal concentration of substrate and enzyme (see Equation S3). From this, the determined normal wild-type minimum and maximum fluxes through the corresponding reaction were adjusted to a perturbed mutant state, and both FVA and MCMC simulations were then run with the goal of analyzing 1) the flux differences through the exchange reactions (import/export of metabolites) of the erythrocyte (as described above in the section “Genetic variation, drug-target interactions, and essential genes”) and 2) significant flux shifts within the internal network. In this way, hypotheses for the altered phenotypic state of the erythrocyte and its impact on the body could be deduced based on the differences of uptake or secretion of metabolites or large-scale internal network changes. For MCMC simulations, significant shifts in the distribution of fluxes were considered (p-value < 0.05). Additional information on MCMC sampling is included in the section entitled “Systems modeling” in S1 Text.

With the kinetic rate law model, we are able to directly integrate the predicted *K*_*m*_ and experimental *K*_*cat*_ values as well as simulate the cell under oxidative or energy load conditions. This detailed model was utilized for the simulations of normal and perturbed G6PD and GAPDH enzymes. Simulation of COMT within the kinetic model was not available due to the current model being limited to core metabolic enzymes. We utilize the model to also understand the erythrocyte’s capability to withstand oxidative stress or increased energy needs and compare wild-type to mutant states. Oxidative stress is simulated as an increase in the rate of NADPH usage, to mirror the fact that a cell under stress requires NADPH to neutralize reactive oxygen species. Energy load is simulated as an increase in the rate of ATP usage. The normal, wild-type cell was first simulated and the maximum oxidative and energy loads were determined for comparison to the mutant state. Integration of the predicted *K*_*m*_ without any change in *K*_*cat*_ was then simulated for the mutant state, to understand if only changes in binding affinity led to a change in maximum tolerable oxidative or energetic load. Finally, changes from predicted *K*_*m*_, experimental *K*_*m*_, and experimental *K*_*cat*_ were fully integrated to investigate the model’s accuracy to the known phenotype.

## Supporting Information

S1 Text Expanded methods and results text and figures detailing GEM-PRO construction, molecular modeling simulations, and systems modeling. (PDF)

S1 Database GEM-PRO of the human erythrocyte and related pharmacogenomics files. Table A: GEM-PRO for *i*AB-RBC-283 (denoted as *i*NM-RBC-283-GP in the main text). Table B: Condensed information on pharmacogenomics and target classification (for molecular and systems modeling ranking) for all enzymes in the RBC model. Table C: Extended information on exonic SNPs found in enzymes of the RBC model. Table D: Extended information on drugs and drug targets found in enzymes of the RBC model. Table E: Extended information on pharmacogenomics found in enzymes of the RBC model. Table F: PDB metadata, structure quality determined by PSQS and PROCHECK, and ranking by resolution and sequence identity. Table G: Homology model template information and structure quality determined by PSQS and PROCHECK.
(XLSX)

S2 Database Parameters used in molecular modeling simulations. Table A-G: COMT ligand parameters for SAM, DNC, TCW, ENT, LDP, ALE, LNR. Table H-I: G6PD ligand parameters for G6P & 6PG. Table J: GAPDH ligand parameters for G3P.
(XLSX)

S3 Database Experimental PDB files and full-length homology models representing each protein in the erythrocyte model. Note that protein complexes are not considered for this analysis, and each file represents a single chain.
(7Z) Click here for additional data file.

## References

[1] Ingelman-Sundberg M. Pharmacogenetics: an opportunity for a safer and more efficient pharmacotherapy. J Intern Med. 2001;250: 186–200. 11555122 10.1046/j.1365-2796.2001.00879.x

[2] Frueh FW, Amur S, Mummaneni P, Epstein RS, Aubert RE, DeLuca TM, et al Pharmacogenomic biomarker information in drug labels approved by the United States food and drug administration: prevalence of related drug use. Pharmacotherapy. 2008;28: 992–998. 10.1592/phco.28.8.992 18657016

[3] Wedlund PJ, de Leon J. Pharmacogenomic testing: the cost factor. Pharmacogenomics J. 2001;1: 171–174. 11908752 10.1038/sj.tpj.6500033

[4] Zielinski DC, Filipp FV, Bordbar A, Jensen K, Smith JW, Herrgard MJ, et al Pharmacogenomic and clinical data link non-pharmacokinetic metabolic dysregulation to drug side effect pathogenesis. Nat Commun. 2015;6: 7101 10.1038/ncomms8101 26055627 PMC4468904

[5] Jamshidi N, Wiback SJ, Palsson B BØ. In silico model-driven assessment of the effects of single nucleotide polymorphisms (SNPs) on human red blood cell metabolism. Genome Res. 2002;12: 1687–1692. 12421755 10.1101/gr.329302 PMC187553

[6] Rajith B C GPD. Path to Facilitate the Prediction of Functional Amino Acid Substitutions in Red Blood Cell Disorders—A Computational Approach. PLoS One. Public Library of Science; 2011;6: e24607 10.1371/journal.pone.0024607 21931771 PMC3172254

[7] Duarte NC, Becker SA, Jamshidi N, Thiele I, Mo ML, Vo TD, et al Global reconstruction of the human metabolic network based on genomic and bibliomic data. Proceedings of the National Academy of Sciences. National Acad Sciences; 2007;104: 1777–1782. 10.1073/pnas.0610772104 PMC1794290 17267599

[8] Bordbar A, Jamshidi N, Palsson BO. iAB-RBC-283: A proteomically derived knowledge-base of erythrocyte metabolism that can be used to simulate its physiological and patho-physiological states. BMC Syst Biol. 2011;5: 110 10.1186/1752-0509-5-110 21749716 PMC3158119

[9] Mardinoglu A, Gatto F, Nielsen J. Genome-scale modeling of human metabolism—a systems biology approach. Biotechnol J. 2013;8: 985–996. 10.1002/biot.201200275 23613448

[10] Hecht M, Bromberg Y, Rost B. Better prediction of functional effects for sequence variants. BMC Genomics. 2015;16 Suppl 8: S1 10.1186/1471-2164-16-S8-S1 26110438 PMC4480835

[11] Jorgensen WL. The many roles of computation in drug discovery. Science. 2004;303: 1813–1818. 15031495 10.1126/science.1096361

[12] Wist AD, Berger SI, Iyengar R. Systems pharmacology and genome medicine: a future perspective. Genome Med. 2009;1: 11 10.1186/gm11 19348698 PMC2651594

[13] Yang R, Niepel M, Mitchison TK, Sorger PK. Dissecting variability in responses to cancer chemotherapy through systems pharmacology. Clin Pharmacol Ther. 2010;88: 34–38. 10.1038/clpt.2010.96 20520606 PMC2941986

[14] Duran-Frigola M, Mosca R, Aloy P. Structural systems pharmacology: the role of 3D structures in next-generation drug development. Chem Biol. Elsevier; 2013;20: 674–684. 10.1016/j.chembiol.2013.03.004 23706634

[15] Xie L, Ge X, Tan H, Xie L, Zhang Y, Hart T, et al Towards structural systems pharmacology to study complex diseases and personalized medicine. PLoS Comput Biol. 2014;10: e1003554 10.1371/journal.pcbi.1003554 24830652 PMC4022462

[16] Agren R, Mardinoglu A, Asplund A, Kampf C, Uhlen M, Nielsen J. Identification of anticancer drugs for hepatocellular carcinoma through personalized genome-scale metabolic modeling. Mol Syst Biol. 2014;10: 721 10.1002/msb.145122 24646661 PMC4017677

[17] Turner RM, Park BK, Pirmohamed M. Parsing interindividual drug variability: an emerging role for systems pharmacology. Wiley Interdiscip Rev Syst Biol Med. 2015;7: 221–241. 10.1002/wsbm.1302 25950758 PMC4696409

[18] Tan H, Ge X, Xie L. Structural systems pharmacology: a new frontier in discovering novel drug targets. Curr Drug Targets. 2013;14: 952–958. 23597016 10.2174/1389450111314090003

[19] Csermely P, Korcsmáros T, Kiss HJM, London G, Nussinov R. Structure and dynamics of molecular networks: a novel paradigm of drug discovery: a comprehensive review. Pharmacol Ther. 2013;138: 333–408. 10.1016/j.pharmthera.2013.01.016 23384594 PMC3647006

[20] Hart T, Xie L. Providing data science support for systems pharmacology and its implications to drug discovery. Expert Opin Drug Discov. 2016;11: 241–256. 10.1517/17460441.2016.1135126 26689499 PMC4988863

[21] Xie L, Xie L, Bourne PE. Structure-based systems biology for analyzing off-target binding. Curr Opin Struct Biol. 2011;21: 189–199. 10.1016/j.sbi.2011.01.004 21292475 PMC3070778

[22] Chang RL, Xie L, Xie L, Bourne PE, Palsson BØ. Drug off-target effects predicted using structural analysis in the context of a metabolic network model. PLoS Comput Biol. 2010;6: e1000938 10.1371/journal.pcbi.1000938 20957118 PMC2950675

[23] Chang RL, Xie L, Bourne PE, Palsson BO. Antibacterial mechanisms identified through structural systems pharmacology. BMC Syst Biol. 2013;7: 102 10.1186/1752-0509-7-102 24112686 PMC3853765

[24] Sorger PK, Allerheiligen SRB, Abernethy DR, Altman RB, Brouwer KLR, Califano A, et al. Quantitative and systems pharmacology in the post-genomic era: new approaches to discovering drugs and understanding therapeutic mechanisms. An NIH white paper by the QSP workshop group. NIH Bethesda; 2011. pp. 1–48.

[25] Zhao S, Iyengar R. Systems pharmacology: network analysis to identify multiscale mechanisms of drug action. Annu Rev Pharmacol Toxicol. 2012;52: 505–521. 10.1146/annurev-pharmtox-010611-134520 22235860 PMC3619403

[26] Gottlieb A, Altman RB. Integrating systems biology sources illuminates drug action. Clin Pharmacol Ther. 2014;95: 663–669. 10.1038/clpt.2014.51 24577151 PMC4029855

[27] Iyengar R, Zhao S, Chung S-W, Mager DE, Gallo JM. Merging systems biology with pharmacodynamics. Sci Transl Med. 2012;4: 126ps7 10.1126/scitranslmed.3003563 22440734 PMC3405973

[28] Brunk E, Mih N, Monk J, Zhang Z, O‘Brien EJ, Bliven SE, et al Systems biology of the structural proteome. BMC Syst Biol. bmcsystbiol.biomedcentral.com; 2016;10: 26 10.1186/s12918-016-0271-6 26969117 PMC4787049

[29] Thorn CF, Klein TE, Altman RB. Pharmacogenomics and bioinformatics: PharmGKB. Pharmacogenomics. 2010;11: 501–505. 10.2217/pgs.10.15 20350130 PMC3098752

[30] Berman HM. The Protein Data Bank. Nucleic Acids Res. 2000;28: 235–242. 10592235 10.1093/nar/28.1.235 PMC102472

[31] Cossum PA. Role of the red blood cell in drug metabolism. Biopharm Drug Dispos. 1988;9: 321–336. 3061491 10.1002/bod.2510090402

[32] Hinderling PH. Red blood cells: a neglected compartment in pharmacokinetics and pharmacodynamics. Pharmacol Rev. 1997;49: 279–295. 9311024

[33] Boudíková B, Szumlanski C, Maidak B, Weinshilboum R. Human liver catechol-O-methyltransferase pharmacogenetics. Clin Pharmacol Ther. 1990;48: 381–389. 2225698 10.1038/clpt.1990.166

[34] Fujii H, Miwa S. Red blood cell enzymes and their clinical application. Adv Clin Chem. 1998;33: 1–54. 10086174 10.1016/s0065-2423(08)60205-x

[35] Zimmerman HJ. Hepatotoxicity: the adverse effects of drugs and other chemicals on the liver Lippincott Williams & Wilkins; 1999.

[36] Fujii H, Miwa S. Other erythrocyte enzyme deficiencies associated with non-haematological symptoms: phosphoglycerate kinase and phosphofructokinase deficiency. Baillieres Best Pract Res Clin Haematol. 2000;13: 141–148. 10916683 10.1053/beha.1999.0062

[37] Sender R, Fuchs S, Milo R. Revised estimates for the number of human and bacteria cells in the body [Internet]. bioRxiv. 2016. p. 036103. 10.1371/journal.pbio.1002533 PMC4991899 27541692

[38] Sherry ST, Ward MH, Kholodov M, Baker J, Phan L, Smigielski EM, et al dbSNP: the NCBI database of genetic variation. Nucleic Acids Res. 2001;29: 308–311. 11125122 10.1093/nar/29.1.308 PMC29783

[39] Amberger JS, Bocchini CA, Schiettecatte F, Scott AF, Hamosh A. OMIM. org: Online Mendelian Inheritance in Man (OMIM), an online catalog of human genes and genetic disorders. Nucleic Acids Res. Oxford Univ Press; 2015;43: D789–D798. 10.1093/nar/gku1205 25428349 PMC4383985

[40] Famiglietti ML, Estreicher A, Gos A, Bolleman J, Géhant S, Breuza L, et al Genetic variations and diseases in UniProtKB/Swiss-Prot: the ins and outs of expert manual curation. Hum Mutat. 2014;35: 927–935. 10.1002/humu.22594 24848695 PMC4107114

[41] Law V, Knox C, Djoumbou Y, Jewison T, Guo AC, Liu Y, et al DrugBank 4.0: shedding new light on drug metabolism. Nucleic Acids Res. 2014;42: D1091–7. 10.1093/nar/gkt1068 24203711 PMC3965102

[42] Günther S, Kuhn M, Dunkel M, Campillos M, Senger C, Petsalaki E, et al SuperTarget and Matador: resources for exploring drug-target relationships. Nucleic Acids Res. 2008;36: D919–22. 17942422 10.1093/nar/gkm862 PMC2238858

[43] Zhou H, Skolnick J. Template-based protein structure modeling using TASSER(VMT.). Proteins. 2012;80: 352–361. 10.1002/prot.23183 22105797 PMC3291807

[44] Roy A, Kucukural A, Zhang Y. I-TASSER: a unified platform for automated protein structure and function prediction. Nat Protoc. 2010;5: 725–738. 10.1038/nprot.2010.5 20360767 PMC2849174

[45] Shen Y, Liu J, Estiu G, Isin B, Ahn Y-Y, Lee D-S, et al Blueprint for antimicrobial hit discovery targeting metabolic networks. Proc Natl Acad Sci U S A. 2010;107: 1082–1087. 10.1073/pnas.0909181107 20080587 PMC2824290

[46] Kazakiewicz D, Karr JR, Langner KM, Plewczynski D. A combined systems and structural modeling approach repositions antibiotics for Mycoplasma genitalium. Comput Biol Chem. 2015; 10.1016/j.compbiolchem.2015.07.007 26271684

[47] Weinshilboum RM, Raymond FA. Inheritance of low erythrocyte catechol-o-methyltransferase activity in man. Am J Hum Genet. 1977;29: 125–135. 848488 PMC1685255

[48] McLeod HL, Fang L, Luo X, Scott EP, Evans WE. Ethnic differences in erythrocyte catechol-O-methyltransferase activity in black and white Americans. J Pharmacol Exp Ther. 1994;270: 26–29. 8035323

[49] Maltête D, Cottard AM, Mihout B, Costentin J. Erythrocytes catechol-o-methyl transferase activity is up-regulated after a 3-month treatment by entacapone in parkinsonian patients. Clin Neuropharmacol. 2011;34: 21–23. 10.1097/WNF.0b013e318205dff7 21164341

[50] Männistö PT, Kaakkola S. Catechol-O-methyltransferase (COMT): biochemistry, molecular biology, pharmacology, and clinical efficacy of the new selective COMT inhibitors. Pharmacol Rev. 1999;51: 593–628. 10581325

[51] Arnold MA, Bartholini G, Black IB, Bloom FE, Brownstein MJ, Conolly ME, et al Catecholamines II Springer Science & Business Media; 2012.

[52] Corvol J-C, Bonnet C, Charbonnier-Beaupel F, Bonnet A-M, Fiévet M-H, Bellanger A, et al The COMT Val158Met polymorphism affects the response to entacapone in Parkinson‘s disease: a randomized crossover clinical trial. Ann Neurol. 2011;69: 111–118. 10.1002/ana.22155 21280081

[53] Rutherford K, Le Trong I, Stenkamp RE, Parson WW. Crystal structures of human 108V and 108M catechol O-methyltransferase. J Mol Biol. 2008;380: 120–130. 10.1016/j.jmb.2008.04.040 18486144

[54] Palma PN, Bonifácio MJ, Loureiro AI, Wright LC, Learmonth DA, Soares-da-Silva P. Molecular modeling and metabolic studies of the interaction of catechol-O-methyltransferase and a new nitrocatechol inhibitor. Drug Metab Dispos. 2003;31: 250–258. 12584150 10.1124/dmd.31.3.250

[55] Rutherford K, Alphandéry E, McMillan A, Daggett V, Parson WW. The V108M mutation decreases the structural stability of catechol O-methyltransferase. Biochim Biophys Acta. 2008;1784: 1098–1105. 10.1016/j.bbapap.2008.04.006 18474266

[56] Wong CF, Kua J, Zhang Y, Straatsma TP, McCammon JA. Molecular docking of balanol to dynamics snapshots of protein kinase A. Proteins. 2005;61: 850–858. 16245317 10.1002/prot.20688

[57] Bolstad ESD, Anderson AC. In pursuit of virtual lead optimization: pruning ensembles of receptor structures for increased efficiency and accuracy during docking. Proteins. 2009;75: 62–74. 10.1002/prot.22214 18781587 PMC2649978

[58] Paulsen JL, Anderson AC. ChemInform Abstract: Scoring Ensembles of Docked Protein: Ligand Interactions for Virtual Lead Optimization. ChemInform. WILEY-VCH Verlag; 2010;41: no–no. 10.1021/ci9003078 PMC2819002 19950979

[59] Cheng LS, Amaro RE, Xu D, Li WW, Arzberger PW, McCammon JA. Ensemble-based virtual screening reveals potential novel antiviral compounds for avian influenza neuraminidase. J Med Chem. 2008;51: 3878–3894. 10.1021/jm8001197 18558668 PMC2652358

[60] Yoon S, Welsh WJ. Identification of a minimal subset of receptor conformations for improved multiple conformation docking and two-step scoring. J Chem Inf Comput Sci. 2004;44: 88–96. 14741014 10.1021/ci0341619

[61] Miller BR III, McGee TD Jr, Swails JM, Homeyer N, Gohlke H, Roitberg AE. MMPBSA. py: an efficient program for end-state free energy calculations. J Chem Theory Comput. ACS Publications; 2012;8: 3314–3321. 10.1021/ct300418h 26605738

[62] Lotta T, Vidgren J, Tilgmann C, Ulmanen I, Melén K, Julkunen I, et al Kinetics of human soluble and membrane-bound catechol O-methyltransferase: a revised mechanism and description of the thermolabile variant of the enzyme. Biochemistry. 1995;34: 4202–4210. 7703232 10.1021/bi00013a008

[63] Chen J, Lipska BK, Halim N, Ma QD, Matsumoto M, Melhem S, et al Functional analysis of genetic variation in catechol-O-methyltransferase (COMT): effects on mRNA, protein, and enzyme activity in postmortem human brain. Am J Hum Genet. 2004;75: 807–821. 15457404 10.1086/425589 PMC1182110

[64] Kirkman HN, Gaetani GD, Clemons EH, Mareni C. Red cell NADP+ and NADPH in glucose-6-phosphate dehydrogenase deficiency. J Clin Invest. 1975;55: 875–878. 235564 10.1172/JCI107998 PMC301824

[65] Beutler E. The molecular biology of G6PD variants and other red cell enzyme defects. Annu Rev Med. 1992;43: 47–59. 1580603 10.1146/annurev.me.43.020192.000403

[66] Mason PJ, Bautista JM, Gilsanz F. G6PD deficiency: the genotype-phenotype association. Blood Rev. 2007;21: 267–283. 17611006 10.1016/j.blre.2007.05.002

[67] Wang X-T, Lam V, Engel PC. Marked decrease in specific activity contributes to disease phenotype in two human glucose 6-phosphate dehydrogenase mutants, G6PDUnion and G6PDAndalus. Hum Mutat. Wiley Online Library; 2005;26: 284–284. 10.1002/humu.9367 16088936

[68] Notaro R, Afolayan A, Luzzatto L. Human mutations in glucose 6-phosphate dehydrogenase reflect evolutionary history. FASEB J. 2000;14: 485–494. 10698963 10.1096/fasebj.14.3.485

[69] Kotaka M, Gover S, Vandeputte-Rutten L, Au SWN, Lam VMS, Adams MJ. Structural studies of glucose-6-phosphate and NADP+ binding to human glucose-6-phosphate dehydrogenase. Acta Crystallogr D Biol Crystallogr. 2005;61: 495–504. 15858258 10.1107/S0907444905002350

[70] Giles NM, Giles GI, Jacob C. Multiple roles of cysteine in biocatalysis. Biochem Biophys Res Commun. 2003;300: 1–4. 12480511 10.1016/s0006-291x(02)02770-5

[71] Kumar P, Henikoff S, Ng PC. Predicting the effects of coding non-synonymous variants on protein function using the SIFT algorithm. Nat Protoc. 2009;4: 1073–1081. 10.1038/nprot.2009.86 19561590

[72] Adzhubei IA, Schmidt S, Peshkin L, Ramensky VE, Gerasimova A, Bork P, et al A method and server for predicting damaging missense mutations. Nat Methods. Nature Publishing Group; 2010;7: 248–249. 10.1038/nmeth0410-248 20354512 PMC2855889

[73] Kisters-Woike B, Vangierdegom C, Müller-Hill B. On the conservation of protein sequences in evolution. Trends Biochem Sci. 2000;25: 419–421. 10973052 10.1016/s0968-0004(00)01631-5

[74] Sirover MA. New insights into an old protein: the functional diversity of mammalian glyceraldehyde-3-phosphate dehydrogenase. Biochim Biophys Acta. 1999;1432: 159–184. 10407139 10.1016/s0167-4838(99)00119-3

[75] Soukri A, Mougin A, Corbier C, Wonacott A, Branlant C, Branlant G. Role of the histidine 176 residue in glyceraldehyde-3-phosphate dehydrogenase as probed by site-directed mutagenesis. Biochemistry. 1989;28: 2586–2592. 2659073 10.1021/bi00432a036

[76] Cook WJ, Senkovich O, Chattopadhyay D. An unexpected phosphate binding site in glyceraldehyde 3-phosphate dehydrogenase: crystal structures of apo, holo and ternary complex of Cryptosporidium parvum enzyme. BMC Struct Biol. 2009;9: 9 10.1186/1472-6807-9-9 19243605 PMC2662861

[77] O’Brien EJ, Monk JM, Palsson BO. Using Genome-scale Models to Predict Biological Capabilities. Cell. 2015;161: 971–987. 10.1016/j.cell.2015.05.019 26000478 PMC4451052

[78] Orth JD, Thiele I, Palsson BØ. What is flux balance analysis? Nat Biotechnol. Nature Publishing Group; 2010;28: 245–248. 10.1038/nbt.1614 PMC3108565 20212490

[79] Bordbar A, Monk JM, King ZA, Palsson BO. Constraint-based models predict metabolic and associated cellular functions. Nat Rev Genet. Nature Publishing Group; 2014;15: 107–120. 10.1038/nrg3643 24430943

[80] Bordbar A, McCloskey D, Zielinski DC, Sonnenschein N, Jamshidi N, Palsson BO. Personalized Whole-Cell Kinetic Models of Metabolism for Discovery in Genomics and Pharmacodynamics. Cell Systems. 2015;1: 283–292. 10.1016/j.cels.2015.10.003 27136057

[81] Jamshidi N, Palsson BØ. Mass action stoichiometric simulation models: incorporating kinetics and regulation into stoichiometric models. Biophys J. 2010;98: 175–185. 10.1016/j.bpj.2009.09.064 20338839 PMC2808481

[82] Shlomi T, Cabili MN, Ruppin E. Predicting metabolic biomarkers of human inborn errors of metabolism. Mol Syst Biol. 2009;5: 263 10.1038/msb.2009.22 19401675 PMC2683725

[83] Schellenberger J, Palsson BØ. Use of randomized sampling for analysis of metabolic networks. J Biol Chem. 2009;284: 5457–5461. 10.1074/jbc.R800048200 18940807

[84] Kim JS, Kim J-Y, Kim J-M, Kim JW, Chung SJ, Kim SR, et al No correlation between COMT genotype and entacapone benefits in Parkinson‘s disease. Neurology Asia. 2011;16 Available: http://search.ebscohost.com/login.aspx?direct=true&db=a9h&AN=67251835&site=ehost-live

[85] Apud JA, Mattay V, Chen J, Kolachana BS, Callicott JH, Rasetti R, et al Tolcapone improves cognition and cortical information processing in normal human subjects. Neuropsychopharmacology. 2007;32: 1011–1020. 17063156 10.1038/sj.npp.1301227

[86] Chong DJ, Suchowersky O, Szumlanski C, Weinshilboum RM, Brant R, Campbell NR. The relationship between COMT genotype and the clinical effectiveness of tolcapone, a COMT inhibitor, in patients with Parkinson‘s disease. Clin Neuropharmacol. 2000;23: 143–148. 10895397 10.1097/00002826-200005000-00003

[87] Glucose-6-phosphate dehydrogenase deficiency. WHO Working Group. Bull World Health Organ. 1989;67: 601–611. 2633878 PMC2491315

[88] Lee B, Scaglia F. Inborn Errors of Metabolism: From Neonatal Screening to Metabolic Pathways Oxford University Press; 2014.

[89] Pretsch W, Favor J. Genetic, biochemical, and molecular characterization of nine glyceraldehyde-3-phosphate dehydrogenase mutants with reduced enzyme activity in Mus musculus. Mamm Genome. 2007;18: 686–692. 17874335 10.1007/s00335-007-9055-z

[90] Seidler NW. GAPDH: Biological Properties and Diversity: Biological Properties and Diversity Springer Science & Business Media; 2012.

[91] Price ND, Schellenberger J, Palsson BO. Uniform sampling of steady-state flux spaces: means to design experiments and to interpret enzymopathies. Biophys J. 2004;87: 2172–2186. 15454420 10.1529/biophysj.104.043000 PMC1304643

[92] Rutherford K, Daggett V. Polymorphisms and disease: hotspots of inactivation in methyltransferases. Trends Biochem Sci. 2010;35: 531–538. 10.1016/j.tibs.2010.03.007 20382027 PMC2928399

[93] Beck DAC, Jonsson AL, Schaeffer RD, Scott KA, Day R, Toofanny RD, et al Dynameomics: mass annotation of protein dynamics and unfolding in water by high-throughput atomistic molecular dynamics simulations. Protein Eng Des Sel. 2008;21: 353–368. 10.1093/protein/gzn011 18411224

[94] van der Kamp MW, Schaeffer RD, Jonsson AL, Scouras AD, Simms AM, Toofanny RD, et al Dynameomics: a comprehensive database of protein dynamics. Structure. 2010;18: 423–435. 10.1016/j.str.2010.01.012 20399180 PMC2892689

[95] Zwier MC, Chong LT. Reaching biological timescales with all-atom molecular dynamics simulations. Curr Opin Pharmacol. 2010;10: 745–752. 10.1016/j.coph.2010.09.008 20934381

[96] Lavecchia A, Di Giovanni C. Virtual screening strategies in drug discovery: a critical review. Curr Med Chem. 2013;20: 2839–2860. 23651302 10.2174/09298673113209990001

[97] Wang L, Wu Y, Deng Y, Kim B, Pierce L, Krilov G, et al Accurate and reliable prediction of relative ligand binding potency in prospective drug discovery by way of a modern free-energy calculation protocol and force field. J Am Chem Soc. 2015;137: 2695–2703. 10.1021/ja512751q 25625324

[98] Bordbar A, Palsson BØ. Moving Toward Genome-Scale Kinetic Models: The Mass Action Stoichiometric Simulation Approach. Functional Coherence of Molecular Networks in Bioinformatics Springer New York; 2012 pp. 201–220.

[99] Zhang Y, Thiele I, Weekes D, Li Z, Jaroszewski L, Ginalski K, et al Three-dimensional structural view of the central metabolic network of Thermotoga maritima. Science. 2009;325: 1544–1549. 10.1126/science.1174671 19762644 PMC2833182

[100] Chang RL, Andrews K, Kim D, Li Z, Godzik A, Palsson BO. Structural systems biology evaluation of metabolic thermotolerance in Escherichia coli. Science. 2013;340: 1220–1223. 10.1126/science.1234012 23744946 PMC3777776

[101] King ZA, Lu J, Dräger A, Miller P, Federowicz S, Lerman JA, et al BiGG Models: A platform for integrating, standardizing and sharing genome-scale models. Nucleic Acids Res. 2016;44: D515–22. 10.1093/nar/gkv1049 26476456 PMC4702785

[102] Maglott D, Ostell J, Pruitt KD, Tatusova T. Entrez Gene: gene-centered information at NCBI. Nucleic Acids Res. 2005;33: D54–8. 15608257 10.1093/nar/gki031 PMC539985

[103] Pruitt KD, Brown GR, Hiatt SM, Thibaud-Nissen F, Astashyn A, Ermolaeva O, et al RefSeq: an update on mammalian reference sequences. Nucleic Acids Res. 2014;42: D756–63. 10.1093/nar/gkt1114 24259432 PMC3965018

[104] Cokelaer T, Pultz D, Harder LM, Serra-Musach J, Saez-Rodriguez J. BioServices: a common Python package to access biological Web Services programmatically. Bioinformatics. 2013;29: 3241–3242. 10.1093/bioinformatics/btt547 24064416 PMC3842755

[105] Kinsella RJ, Kähäri A, Haider S, Zamora J, Proctor G, Spudich G, et al Ensembl BioMarts: a hub for data retrieval across taxonomic space. Database. 2011;2011: bar030 10.1093/database/bar030 21785142 PMC3170168

[106] Menon R, Roy A, Mukherjee S, Belkin S, Zhang Y, Omenn GS. Functional implications of structural predictions for alternative splice proteins expressed in Her2/neu-induced breast cancers. J Proteome Res. 2011;10: 5503–5511. 10.1021/pr200772w 22003824 PMC3230717

[107] Jaroszewski L, Pawlowski K, Godzik A. Multiple Model Approach: Exploring the Limits of Comparative Modeling. J Mol Med. Springer-Verlag; 4: 294–309.

[108] Laskowski RA, MacArthur MW, Moss DS, Thornton JM. ıt PROCHECK: a program to check the stereochemical quality of protein structures. J Appl Crystallogr. 1993;26: 283–291.

[109] Yip YL, Famiglietti M, Gos A, Duek PD, David FPA, Gateau A, et al Annotating single amino acid polymorphisms in the UniProt/Swiss-Prot knowledgebase. Hum Mutat. 2008;29: 361–366. 10.1002/humu.20671 18175334

[110] Gaulton A, Bellis LJ, Bento AP, Chambers J, Davies M, Hersey A, et al ChEMBL: a large-scale bioactivity database for drug discovery. Nucleic Acids Res. 2012;40: D1100–7. 10.1093/nar/gkr777 21948594 PMC3245175

[111] Whirl-Carrillo M, McDonagh EM, Hebert JM, Gong L, Sangkuhl K, Thorn CF, et al Pharmacogenomics knowledge for personalized medicine. Clinical Pharmacology & Therapeutics. Wiley Online Library; 2012;92: 414–417. 22992668 10.1038/clpt.2012.96 PMC3660037

[112] Chang A, Scheer M, Grote A, Schomburg I, Schomburg D. BRENDA, AMENDA and FRENDA the enzyme information system: new content and tools in 2009. Nucleic Acids Res. 2009;37: D588–92. 10.1093/nar/gkn820 18984617 PMC2686525

[113] Liu T, Lin Y, Wen X, Jorissen RN, Gilson MK. BindingDB: a web-accessible database of experimentally determined protein–ligand binding affinities. Nucleic Acids Res. 2007;35: D198–D201. 17145705 10.1093/nar/gkl999 PMC1751547

[114] Mahadevan R, Schilling CH. The effects of alternate optimal solutions in constraint-based genome-scale metabolic models. Metab Eng. 2003;5: 264–276. 14642354 10.1016/j.ymben.2003.09.002

[115] Lang PT, Brozell SR, Mukherjee S, Pettersen EF, Meng EC, Thomas V, et al DOCK 6: Combining techniques to model RNA–small molecule complexes. RNA. 2009;15: 1219–1230. 10.1261/rna.1563609 19369428 PMC2685511

[116] Case DA, Babin V, Berryman J, Betz RM, Cai Q, Cerutti DS, et al Amber 14 University of California; 2014; Available: https://orbilu.uni.lu/handle/10993/16614

[117] Frisch MJ, Trucks GW, Schlegel HB, Scuseria GE, Robb MA, Cheeseman JR, et al. 01; Gaussian, Inc. Wallingford, CT. 2004;

[118] Martins SA, Sousa SF, Ramos MJ, Fernandes PA. Prediction of Solvation Free Energies with Thermodynamic Integration Using the General Amber Force Field. J Chem Theory Comput. 2014;10: 3570–3577. 10.1021/ct500346y 26588320

[119] Almaas E, Kovács B, Vicsek T, Oltvai ZN, Barabási A-L. Global organization of metabolic fluxes in the bacterium Escherichia coli. Nature. 2004;427: 839–843. 14985762 10.1038/nature02289

