# Supplementary material for: A Multi-scale Computational Platform to Mechanistically Assess the Effect of Genetic Variation on Drug Responses in Human Erythrocyte Metabolism
Source: PLoS Comput Biol. 2016 Jul 28;12(7):e1005039. doi: 10.1371/journal.pcbi.1005039 (PMC4965186; doi:10.1371/journal.pcbi.1005039)
Supplement: S1 Text — (PDF) [file pcbi.1005039.s001.pdf]

A Multi-scale Computational Platform to Mechanistically Assess the Effect of Genetic Variation on Drug Responses in Human Erythrocyte Metabolism

Supplementary Information

Nathan Mih[a, †], Elizabeth Brunk*[b, †], Aarash Bordbar[b], Bernhard O. Palsson*[b,c]

* Correspondence should be addressed to: E.B. (ebrunk@ucsd.edu); B.O.P. (palsson@ucsd.edu)

[a] Bioinformatics and Systems Biology Graduate Program, University of California, San Diego, CA 92093

[b] Department of Bioengineering, University of California, San Diego, CA 92093

[c] Department of Pediatrics, University of California, San Diego, CA 92093

[†] Authors contributed equally

Table of contents

- Methods
  - GEM-PRO construction
    - Identifier mapping
    - Homology modeling
    - QC/QA procedure
        - Maximum coverage of wild-type amino acid sequence
        - Resolution quality of the protein structure
        - Assessment of composition of secondary structural features
    - Model refinement
  - Molecular modeling simulations
    - Ligand parameterization & molecular modeling
      - COMT
      - G6PD
      - GAPDH
    - Molecular dynamics
      - COMT
      - G6PD
      - GAPDH
    - Ensemble docking and clustering of poses
      - COMT
      - G6PD
      - GAPDH
    - Binding energy calculations
  - Systems modeling
    - Perturbed state simulations
    - Drug inhibitor simulations
    - Biomarker detection
    - Markov chain Monte Carlo (MCMC) sampling
- Results
  - GEM-PRO coverage
  - Pharmacogenomics data mapping and classification
  - Molecular modeling simulations
    - COMT
    - G6PD
    - GAPDH
- Additional References

Methods

GEM-PRO construction

The construction of GEM-PROs has previously been detailed in [1–4]. Briefly, the main goal is to take a genome-scale metabolic model in SBML format and correctly map all gene identifiers and known protein complexes to 1) all experimentally determined protein structures in the Protein Data Bank (PDB) and 2) generate homology models for those that have no experimental representation. We have developed a semi-automated pipeline to construct and update GEM-PROs, outlined in [5]. Here, we generate a GEM-PRO of the human erythrocyte, based on the proteomically-derived metabolic reconstruction, *i*AB-283-RBC [6]. The GEM-PRO version of this model will be referred to as *i*NM-283-RBC-GP.

The general procedure for mapping protein structural data to a metabolic network is as follows: (i) establish links between genes in the model, gene transcript links in RefSeq, identifiers in the UniProt sequence database and available PDB structures in the Protein Data Bank, (ii) generate homology models for proteins without any experimental structures, (iii) assess the quality of all available (experimental and homology) structures to determine the most representative structures that can be used for molecular modeling simulations, and (iv) organize all available information into reproducible, queryable, and easily updated database. One additional consideration for this system, as well as for all eukaryotic models, is that alternatively spliced transcripts require special attention, as detailed in Figure A.

Identifier mapping

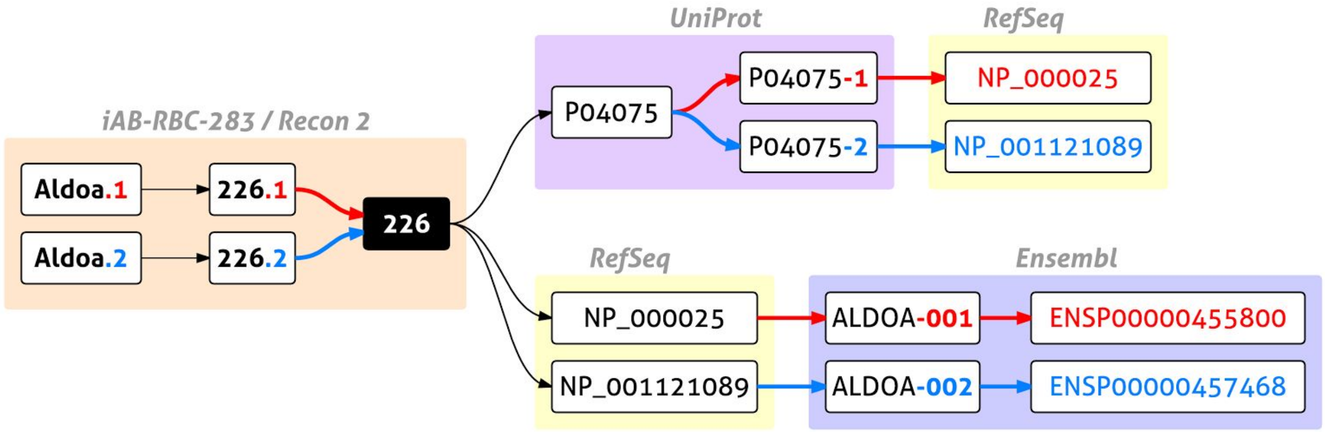

Figure A: Ideal workflow for mapping gene identifiers to the UniProt, RefSeq, and Ensembl databases. The example shown here is for model gene name Aldoa (fructose-bisphosphate aldolase A), with Entrez gene ID 226. There are two isoforms annotated for in both the erythrocyte and human models, 226.1 and 226.2. Taking the gene ID without the Recon 2 isoform IDs, we are able to map it directly to the UniProt database which contains 2 annotated isoforms, and then map them back to the Entrez gene IDs. A separate workflow maps the gene ID (without isoform ID) to the RefSeq database, and transcript names are utilized to assign isoforms. If information from the first workflow does not match that of the second, mapping is subject to manual review.

Homology modeling

The I-TASSER4.0 package [7] was obtained and utilized for manual generation of homology models that were not available in existing databases, such as the Protein Model Portal or SUNPRO [8,9]. The quality of the generated models was calculated using PSQS (available at: http://smb.slac.stanford.edu/jcsg/QC/) [10], as well as PROCHECK (available at: http://www.ebi.ac.uk/thornton-srv/software/PROCHECK/) [11]. Furthermore, the C-score provided by I-TASSER is included, which indicates the level of confidence for a model on a scale of

$[0, 1]$, with scores closer to 1 indicating high confidence. For all results on homology model quality see Table G in S1 Database.

QC/QA procedure

In order to rank order all PDB files for a given gene, we created a score in order to do so:

$$S_{PDB} = S_{SI} + S_{res} + S_{SS}$$

[Equation A]

where S_{PDB} refers to the total quality score of a single PDB file, which is based on a sum of scaled values from $[0, 1]$ (with a score of 1 representing the top ranked structure of the property for a gene) of the percent sequence identity (S_{SI}), the resolution (S_{res}) of the crystallographic structure, and, for cases where I-TASSER homology models were available, the similarity (Jaccard similarity score) of secondary structural features between the PDB structure and its corresponding homology model (S_{SS}). Furthermore, we also separately consider the overall completeness of resolved residues in the protein (if there are gaps in resolved amino acids within the structure) and the difference in the percent α/β secondary structure elements compared to the I-TASSER homology model.

Maximum coverage of wild-type amino acid sequence

The first consideration of the quality of an experimental structure is simply how similar its encoded sequence matches the wild-type sequence of the gene product (S_{SI}). For PDB structures with multiple chains (of which may or may not be encoded by the same gene), we simply rank the PDB file via the alignment score of the chain corresponding to the gene. If multiple chains correspond to one gene, the highest aligning chain was taken as the percent sequence identity. All sequence alignments were conducted by first extracting only resolved amino acids available in the PDB structure (which differ from what is reported in the SEQRES field) using Biopython and then utilizing the EMBOSS needle package for pairwise sequence alignment between these amino acids and the canonical protein sequence [12,13].

Furthermore, we assess completeness or the degree of missing or unresolved fragments of the protein. This identifies whether there are major sequential gaps in the protein structure, which may require further homology modeling. While S_{SI} will undoubtedly rank structures such as these with lower scores, we are also interested in knowing why a PDB structure has a lower ranking compared to others. Structures with observed gaps greater than two residues (ignoring gaps within 10 residues of the N or C termini) are marked as lower quality structures. If an unresolved region of a protein has less than two sequential residues missing, we have carried out standard molecular modeling techniques to minimally modify and insert the missing residue. Otherwise, we perform homology modeling to fill in the larger gaps in sequence.

Resolution quality of the protein structure

The second metric, S_{res}, for ranking PDB structures is based on the resolution, which is a descriptor of the degree of confidence in the resolved atomic coordinates (in Å) of all heavy atoms (for NMR structures, we consider the first member of the ensemble). A higher resolution indicates that a smaller Angstrom distance between atoms can be seen. The resolution for each structure was obtained from the header section of the PDB file, and cross-referenced with the entry from the PDB website.

Assessment of composition of secondary structural features

The final metric for ranking PDB structures, S_{SS}, assesses the similarity in the percent alpha helix/beta sheet composition of a PDB structure and an available homology model. We utilize an implementation of the Jaccard similarity score to measure the similarity of location and length of alpha helices and beta sheets. We reason that if a homology model has been generated utilizing the PDB structure or highly related structure as a template, it has gone through several refinements to become an energetically more favorable structure.

It is important to note that this quality assessment pipeline has been designed to identify which structures are of high confidence and suitable for molecular modeling, and all available PDB structures for a given gene are still stored in the master GEM-PRO data frame.

Model refinement

Once the available experimental structures have been rank ordered, we obtain three sets of structures, some of which may require additional refinement. The first set are experimental structures that meet all criteria above. The second set contains structures that differ from the wild-type sequence only by point mutations, and are used as input for this refinement step in order to revert the PDB sequence to the wild-type sequence and fill in missing parts of the protein that do not exceed 1 residue per gap. The third set contains experimental structures that are ranked lower than the homology models, due to not meeting the cutoffs as outlined.

For the second set, these were corrected initially using the Biopython structural bioinformatics module [12] by altering the sequence of the PDB file. First, the R-group atoms are stripped, leaving only the peptide backbone atoms of the given residue. Next, the amino acids present in the sequence of interest are filled in, and the PDB file is then passed through the AMBERtools suite of programs (AMBER14) to fill in the heavy atoms of the newly changed amino acid [14]. The structure is then minimized with a steepest descent minimization for 10,000 cycles to relieve any overlapping van der Waals interactions. A final QC/QA step was taken by aligning the final wild-type PDB structure to the desired sequence to ensure a final correct structure. In a small handful of cases, the automatic mutation pipeline failed to mutate the correct residue due to inconsistent residue numbering in the PDB file or the use of insertion codes. For these cases, the PDB file was manually altered and minimized.

To summarize, using the above identifier mapping, QC/QA, and refinement pipelines, the updated GEM-PRO models provide representative, high-quality protein structures for a each gene product in the metabolic model. The overall quality of the selected experimental and homology-based structures is detailed in Tables A, F, & G in S1 Database.

Molecular modeling simulations

For each of the high priority protein targets identified in the main text, we performed substrate docking and molecular dynamics simulations using the following protocol: (i) for both wild-type and variant structures, the native metabolites and drug molecules known to inhibit or bind to a given protein were docked to the crystallographic or homology models; (ii) molecular dynamics (MD) simulations were carried out on both apo (substrate-free) and holo (substrate and cofactor bound) enzymes to generate an ensemble of structures, which represent multiple possible conformations of the structure; (iii) a set of simulation frames were extracted as an ensemble of structures, representing different conformational states of the protein which occur on longer time scales (on the order of 100 nanoseconds), and used as (iv) individual structures in docking simulations, where binding modes were clustered to attain the most representative binding modes of a given substrate or drug; (v) molecular residence times of the most representative binding orientations of a metabolite or drug were tested by performing 10 nanoseconds of MD; (vi) for metabolites/drugs with high binding fidelity, changes in binding free energy in the wild type versus SNP variant structures were computed using Molecular Mechanics/Generalized Born Surface Area and Molecular Mechanics/Poisson−Boltzmann Surface Area (MM-GBSA, MM-PBSA) methods. These computations were compared with higher accuracy computations, such as thermodynamic integration (TI).

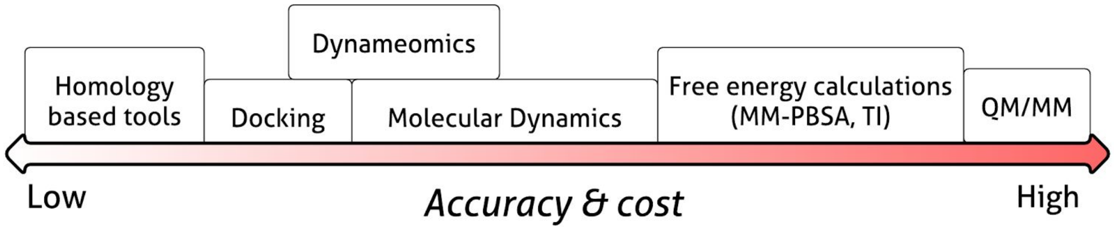

Figure B: Spectrum of molecular modeling tools that will be evaluated for use at the genome-scale. On the low end of accuracy and computational cost are sequence and structure homology-based methods to predict drug binding and the consequences of variation [15]. Docking methods move up on the scale of computational cost, but provide extra information on ligand binding and atom-atom interactions [16]. Molecular dynamics tools allow for the prediction of conformational changes in 3D space and require a much larger amount of computational time [17]. The Dynameomics database provides a stepping stone before MD by providing access to previously generated MD simulations of protein structures along with significant SNPs [18]. Finally, at the high end of accuracy and cost scale are free energy calculations [19,20] which can provide detailed parameters as input to COBRA methods or kinetic cell models, and QM/MM methods to inspect changes at the quantum level and understand rates of catalysis [21].

As a result of the wide spectrum in accuracy and computational cost of different molecular modeling techniques (Figure B), each step of this structural systems pharmacology pipeline has been designed to act independently, for example - if a crystal structure or homology model of a mutant provides enough evidence for a change of drug interaction due to the variant, we do not propose the need to proceed on with molecular dynamics or further calculations but instead towards experimental work. However, in cases where significant associations are known and docking methods are unable to replicate the drug binding utilizing only a single crystal structure (see the section on catechol-O-methyltransferase for an explicit example), we look towards more computationally intensive tools to provide a dynamic view of the effects of variation.

Ligand parameterization & molecular modeling

Force field parameters for all ligands and cofactors for each of the enzymes studied are detailed below. Calculations of optimized geometries and force constants for any molecules without existing parameter sets were generating utilizing Gaussian 09 in the gas phase at the HF/6-31G* level and charges were fitted with the RESP technique [22]. The original molecular specification files were obtained from the PDB Ligand Expo [23] if

available, or from PubChem [24]. Once optimized geometries were calculated, charges and atom types were assigned with the AMBER GAFF using antechamber to be compatible with simulations using the 99SB force field [25].

COMT

Table A: Substrates, cofactors, and drugs parameterized in this study for COMT.

**Molecule**	**Abbreviation**	**2D representation**	**Function**
Magnesium	MG	N/A	Required for methyltransferase activity
S-adenosyl-methionine	SAM	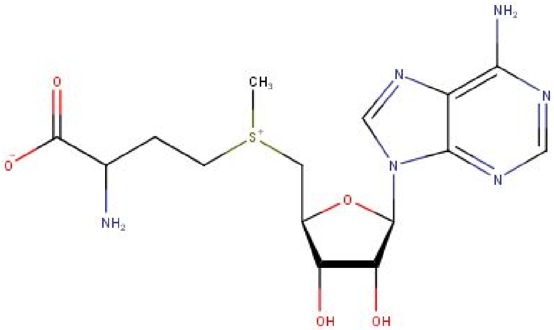	Methyl group is transferred from SAM to the bound catecholamines
Dinitrocatechol	DNC	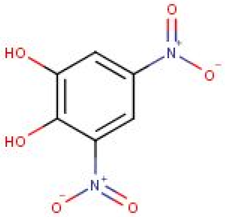	Analog to inhibitors
Tolcapone	TCW	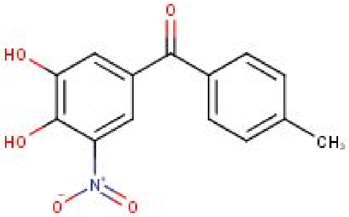	FDA approved inhibitor
Entacapone	ENT	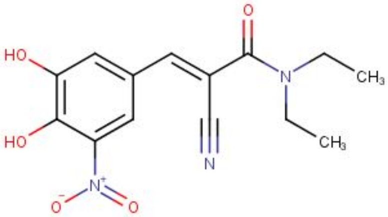	FDA approved inhibitor
Dopamine	LDP	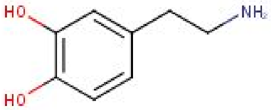	Native metabolite

Epinephrine	ALE	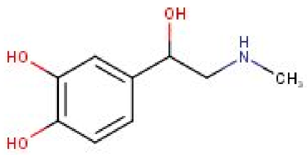	Native metabolite
Norepinephrine	LNR	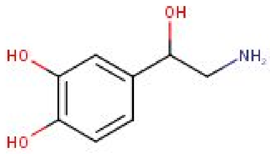	Native metabolite

PDB IDs 3BWM and 3BWY represent the wild-type and mutant (dbSNP ID rs4680) forms of COMT, respectively [26]. The wild-type sequence for COMT was designated per the canonical sequence from UniProt (entry P21964) as well as previous studies which have inspected the different activity levels of COMT [27]. These structures both contain 3 substrates: dinitrocatechol (DNC), S-adenosyl-methionine (SAM), and a magnesium ion. DNC is an inhibitor of COMT, and is an analog to the popular drugs tolcapone (TCW) and entacapone (ENT). SAM is the source of the transferred methyl group, which functions to inactivate the native metabolites of COMT, dopamine (LDP), epinephrine (ALE), norepinephrine (LNR), and others not inspected in this study. All small molecules are defined above in Table A and parameters used in simulations in Tables A-G in S2 Database.

Parameters for all drugs (TCW, ENT, DNC), native metabolites (LDP, ALE, LNR), and cofactors (SAM) were manually generated by optimizing the molecular structure with Gaussian and assigning charges and atom types with the AMBER GAFF using antechamber.

G6PD

Table B: Substrates, cofactors, and drugs parameterized in this study for G6PD.

Molecule	Abbreviation	2D representation	Function
$NADP^+$	NPD	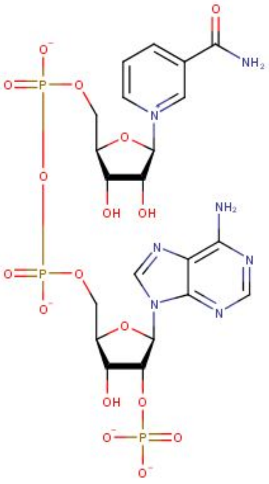	2 NADP+ molecules exist in the monomeric form of G6PD. One functions as a cofactor, and the other functions as a structural subunit [28].

Glucose 6-phosphate	G6P	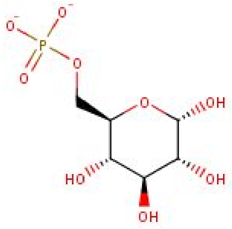	Native metabolite
6-phosphogluconolactone	6PG	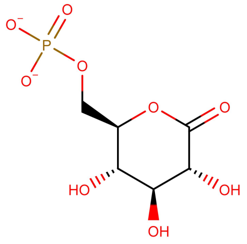	Product of enzyme catalysis

PDB ID 2BH9 [28] was utilized as the wild-type structure of G6PD. Its amino acid sequence matched the canonical UniProt entry P11413, except for an engineered mutation of His27Val and a deletion of the first 25 N-terminal residues. Kotaka et al. showed that this structure had similar kinetic properties to the wild-type enzyme, and these modifications were for the purpose of obtaining a higher quality crystal structure. This entry is complexed with two molecules of $NADP^+$, one of which acts as a coenzyme and the other as a structural unit. The His27Val mutation was manually corrected back to a histidine and protonation states assigned at pH 7 using the PROPKA software available on the PDB2PQR server [29].

Parameters for the bound cofactor, $NADP^+$, were obtained from the AMBER parameter database (available at: http://sites.pharmacy.manchester.ac.uk/bryce/amber). The parameter set contributed by Ryde was utilized [30]. Parameters for glucose 6-phosphate (G6P) and 6-phosphogluconolactone (6PG) were manually generated by optimizing the molecular structure with Gaussian 09 and assigning charges and atom types with the AMBER GAFF using antechamber as described above.

Furthermore, PDB ID 2BHL [28] is complexed with G6P, but without $NADP^+$ as the electron density maps showed less concrete evidence of full occupancy of the cofactor. This structure was utilized in calculating RMSDs and distances to binding residues for G6P in later docking simulations.

The mutant inspected in this study known as the “Andalus” SNP (Arg454His; dbSNP ID rs137852324), was manually mutated from the utilized wild-type structure and minimized with a steepest descent minimization for 10,000 cycles to relieve any overlapping van der Waals interactions as per our model refinement pipeline. Correct protonation states of the residues were again were assigned utilizing the PROPKA software. All small molecules are defined above in Table B and parameters used in simulations for G6P and 6PG are in Tables H & I in S2 Database.

GAPDH

Table C: Substrates, cofactors, and drugs parameterized in this study for GAPDH.

Molecule	Abbreviation	2D representation	Function
NAD+	NAD	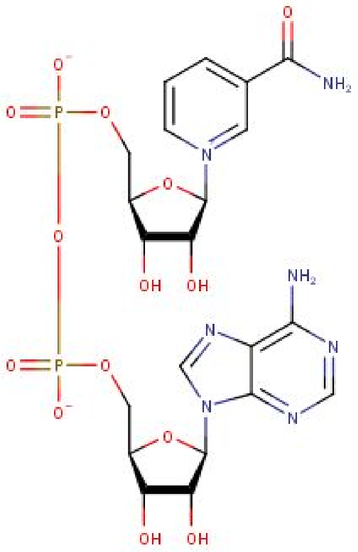	Cofactor
Glyceraldehyde 3-phosphate	G3P	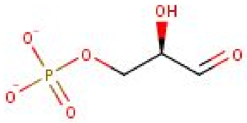	Native metabolite

PDB ID 1U8F [31] was chosen as the experimental model to use as it had a 100% sequence alignment to the UniProt sequence (P04406). The missense mutation, Lys309Asn (dbSNP ID rs11549334) was chosen per initial results from Polyphen and SIFT [32,33] which indicated the highly conserved nature of this residue and noted potentially damaging effects. We manually modeled this mutation and minimized the resulting structure with our model refinement pipeline.

Parameters for the bound cofactor, NAD^+, were obtained from the AMBER parameter database (available at: http://sites.pharmacy.manchester.ac.uk/bryce/amber). The parameter set contributed by Walker, et al. was utilized [34] Parameters for glyceraldehyde 3-phosphate (G3P) were manually generated by optimizing the molecular structure with Gaussian 09 and assigning charges and atom types with the AMBER GAFF using antechamber as described above.

Unfortunately, none of the available experimental structures for GAPDH included the binding of G3P substrate. As such, for understanding the correct binding position of G3P, we utilized distances from known interacting residues found in literature [35–37]. All small molecules are defined above in Table C and parameters used in simulations for G3P are in Table J in S2 Database.

Molecular dynamics

For all simulations, atom names of small molecules were first confirmed to match those available in downloaded or manually generated parameter sets. Single-chain simulations were carried out for all three enzymes, as the location of binding sites are not between dimerization sites. Apo or other cofactor unbound

states of enzymes were generated by manually deleting the cofactors using the Chimera software suite [38] and saved as PDB structure files. All final structure files were then converted to AMBER topology files, assigned charges, atom types, and solvated as noted in the main text.

COMT

Table D: Simulation information for COMT.

**Cofactor bound state**	**WT ID**	**SNP ID**	**Simulation time**	**Number of extracted frames for ensemble docking**
Apo<br>*no cofactors*	WT-apo	SNP-apo	75 ns	500
Apo + SAM<br>*with S-adenosyl methionine*	WT-sam	SNP-sam	75 ns	500
Holo<br>*with S-adenosyl methionine and magnesium*	WT-holo	SNP-holo	113 ns	750

G6PD

Table E: Simulation information for G6PD.

**Cofactor bound state**	**WT ID**	**SNP ID**	**Simulation time**	**Number of extracted frames for ensemble docking**
Apo<br>*no cofactors*	WT-apo	SNP-apo	113 ns	750
Holo<br>*with* $NADP^+$	WT-holo	SNP-holo	113 ns	750

GAPDH

Table F: Simulation information for GAPDH.

**Cofactor bound state**	**WT ID**	**SNP ID**	**Simulation time**	**Number of extracted frames for ensemble docking**
Apo<br>*no cofactors*	WT-apo	SNP-apo	113 ns	750
Holo<br>*with* NAD^+	WT-holo	SNP-holo	113 ns	750

Ensemble docking and clustering of poses

The resulting frames obtained from the molecular dynamics simulations for each protein were collected for use in ensemble docking. The procedure for ensemble docking was to treat each frame in the trajectory as a single rigid structure to use for flexible ligand docking [39,40]. All previously assigned charges were utilized in docking simulations by loading the previously generated AMBER parameter/topology files into Chimera using the MD Movie function. The included Dock Prep program was then run on each frame, and finally DOCK6 was run with the the previously parameterized substrates and defined binding sites (see protein-specific information below) [38,41]. All binding sites used in this study are reported in the tables below with the original PDB residue numbering. Only the highest scoring docking pose was taken for each frame that was docked to for subsequent clustering.

To cluster the docked poses, we took a simple distance based approach to understand the “correctness” of ligand position to known catalytic residues. To do so, for each protein, three atom-atom pairs of distances between the ligand and receptor were chosen based on known binding sites in literature. Then, each of these pairs were measured in all docked poses to the ensemble. The results of these distances were then clustered using the mean shift clustering method from the Scipy Python package [42]. The mean shift clustering algorithm is similar to K-means in that centroids are chosen to be as close to the mean as possible for a group of points, however, the number of clusters does not have to be chosen beforehand. As a result, this allowed for a quick way to understand if there was a group of docked ligands that was close to the correct atom-atom distance for each of the three pairs. Subsequently, for each of these pairs, we designated a “correct” label for those that were in the cluster closest to the interacting residue, and then created groups that indicated if all three pairs were “correct”, if only two were, and so on (see Figure H for a visual representation). This allowed us to quickly cluster the hundreds of predicted binding poses from ensemble docking. Finally, a representative from each of these groups was chosen based on manual inspection of the group; frequently, similar poses were contained in the same groups except for the cases when all three pairs were “incorrect”, which would show (presumably incorrect) binding poses at different sites of the enzyme. This representative for the group was used in later binding free energy calculations. A minimum of 3 groups were chosen for this study, in order to average the binding free energy results for plausible binding poses within the enzyme.

COMT

Table G: Residues surrounding the inhibitor dinitrocatechol within the wild-type COMT structure (PDB ID 3BWM). The numbering corresponds to the UniProt isoform sequence P21964-2 (the soluble version of COMT). The three residues and the respective atoms chosen for clustering are also listed, as well as the closest interacting atom on DNC.

**Amino acid**	**PDB residue number (3BWM, 3BWY)**	**Atom used for clustering**	**Substrate atom interaction (DNC)**	**Function**
Tryptophan	38	**CH2**	**O5**	Closest to selected substrate atom
Lysine	144	**NZ**	**O3**	Annotated binding site; catalytic base in the nucleophilic methyl transfer reaction [43]
Asparagine	170	N/A	N/A	Annotated binding site (UniProt entry P21964)
Glutamic acid	199	**OE2**	**O1**	Annotated binding site (UniProt entry P21964)

G6PD

Table H: Binding site residues for G6PD. The three residues and the respective atoms chosen for clustering are listed, as well as the closest interacting atom of the substrate, G6P, from PDB entry 2BHL.

**Amino acid**	**PDB residue number (2BH9)**	**Atom used for clustering**	**Substrate atom interaction (G6P)**	**Function**
Lysine	171	N/A	N/A	Annotated binding site (UniProt entry P11413)
Histidine	201	**NE2**	**P**	Closest to selected substrate atom
Lysine	205	N/A	N/A	Essential for substrate catalysis [44]
Glutamic acid	239	**OE2**	**O4**	Annotated binding site (UniProt entry P11413)
Asparagine	258	N/A	N/A	Annotated binding site (UniProt entry P11413)
Histidine	263	**NE2**	**O5**	Proton acceptor (UniProt entry P11413)
Lysine	360	N/A	N/A	Annotated binding site (UniProt entry P11413)

Arginine	365	N/A	N/A	Annotated binding site (UniProt entry P11413)
Glutamine	395	N/A	N/A	Annotated binding site (UniProt entry P11413)

GAPDH

Table I: Binding site residues for GAPDH. The three residues and the respective atoms chosen for clustering are listed (from PDB entry 1U8F), as well as the closest interacting atom of the substrate, G3P. These residues were chosen based on literature reports [35–37]

Amino acid	PDB residue number (1DC4)	PDB residue number (1U8F)	UniProt residue number	Atom used for clustering	Substrate atom interaction (G3P)	Function
Cysteine	149	152	152	**SG**	**O1**	Nucleophile (UniProt entry P04406)
Histidine	176	179	179	**NE2**	**P**	Activates thiol group during catalysis (UniProt entry P04406)
Threonine	179	182	182	N/A	N/A	Annotated binding site (UniProt entry P04406)
Arginine	231	234	234	N/A	N/A	Annotated binding site (UniProt entry P04406)
NAD+	N/A	336	N/A	**O7N**	**O2**	Hydride ion acceptor

Binding energy calculations

Following the clustering of docked poses of the ligand of interest to wild-type and mutant proteins, binding free energy calculations were carried out utilizing Molecular Mechanics-Poisson Boltzmann Surface Area (MM-PBSA) and Molecular Mechanics-Generalized Born Surface Area (MM-GBSA) methods, as well as the more computationally expensive Thermodynamic Integration (TI) technique. MM-GBSA/MM-PBSA methods have previously been used in computational mutagenesis studies to understand the importance of certain residues for ligand or protein-protein binding binding [45–50], and have generally shown good agreement with trends in experimental datasets.

For MM-GBSA/MM-PBSA calculations, the single trajectory approach was used by first conducting a 10 ns simulation of each of the complexes determined from the cluster analysis. All previously generated ligand or substrate parameters were utilized as described in the Molecular Dynamics section above and the simulation procedure for these complexes was identical to as described in the main text. Once these simulations were complete, every 100 frames was extracted from the trajectory for a total of 60 to 80 frames as input for the MMPBSA.py script available in AMBER14 [51]. The corresponding ligand and receptor parameter/topology files were extracted from the complex simulations. Because of the influence different input parameters can have in MM-GBSA/MM-PBSA calculations [19], a number of GB models were tested for consistency. ΔG values reported in the main text are only from MM-PBSA calculations. MM-PBSA parameters were set with a SASA-based model for non-polar solvation free energy calculations (inp=1) and atomic radii were used from the AMBER generated parameter/topology files (radiopt=0), which were originally set to the bondi radii set. Contributions from entropy were not considered in the current study.

As described in the main text, thermodynamic integration (TI) calculations were calculated utilizing the SANDER module within AMBER14. Ligand unbound states for wild-type and mutant enzymes were taken from equilibrated structures from the original (apo) MD simulations detailed above. Ligand bound states were taken

from manual inspection of the docked poses and also selected for based on the lowest predicted binding free energy from MM-PBSA calculations. Furthermore, for COMT, since the mutant crystal structure was available, “reverse” TI was done by setting the mutant structure as state 0 and the wild-type as state 1.

Systems modeling

In order to be predictive of the wide range of effects that drugs or sequence variants can have on a protein, and not approach them as having a binary on-off effect, complementation with structure-based methods here provides quantitative information on changes to the corresponding reactions. Constraint-based modeling approaches aim to understand the allowable solution space of a metabolic network under specified constraints (e.g. uptake and secretion rates of transport reactions), given a cellular objective function (e.g. cellular growth, ATP production, ROS response, etc). By integrating a calculated ratio of fluxes of the mutant enzyme, determined as a function of the predicted ΔΔG, we can directly use the information gained from structural calculations in constraint-based modeling methods.

Perturbed state simulations

Assuming Michaelis-Menten kinetics of a simple reaction, we can calculate the free energy of binding ΔG by [52]

$$\Delta G = - RT \cdot ln(K_{eq}) = RT \cdot ln(K_d)$$

[Equation B]

and also, assuming that the rate of substrate dissociation is much greater than the rate of product formation (K_{cat}),

$$\Delta G = - RT \cdot ln(K_{eq}) = RT \cdot ln(K_d) = RT \cdot ln(K_M)$$

we can calculate the difference of binding free energies between wild-type and mutant proteins to a ligand by Equation C.

$$\Delta\Delta G_{WT-SNP} = \Delta G_{WT} - \Delta G_{SNP} = RT \cdot ln(K_M^{WT}) - RT \cdot ln(K_M^{SNP}) = RT \cdot ln(\frac{K_M^{WT}}{K_M^{SNP}})$$

[Equation C]

As a result, we can directly calculate the relative difference in the binding free energy to a ratio of Michaelis constants, each of which are inversely related to binding affinity. In order to relate to the flux through a reaction, we consider a flux J, the rate of the forward reaction v_F, and the rate of the reverse reaction v_R, where

$$J = v_F - v_R$$

and assuming irreversibility of the reverse reaction v_R while defining v_F in terms of standard Michaelis-Menten kinetics,

$$J = v_F = \frac{V max\,[S]}{K_M}$$

where V_{max} is the maximal rate of catalysis through the enzyme and $[S]$ the substrate concentration. This defines the flux of a reaction as equal to the rate of the forward reaction. By determining the wild-type range of flux through the reaction by flux variability analysis (FVA) as described in the main text, we can designate the flux through a perturbed reaction with the mutant enzyme, assuming equal V_{max} and $[S]$, with,

$$\frac{J^{SNP}}{J^{WT}} = \frac{v_F^{SNP}}{v_F^{WT}} = \frac{K_M^{WT}}{K_M^{SNP}}$$

$$J^{SNP} = J^{WT} \cdot \frac{K_M^{WT}}{K_M^{SNP}}$$

[Equation D]

Finally, this allows us to adjust both the minimum and maximum fluxes of the wild-type reaction to simulate the perturbed state of the cell based on the mutation.

Drug inhibitor simulations

For the purposes of understanding drug inhibition, we can use similar assumptions to the above while adding the effects of a competitive inhibitor. Doing so, it is simple to relate the flux of the inhibited mutant enzyme to the flux of the inhibited wild-type enzyme:

$$J^{SNP+I} = J^{WT+I} \cdot \frac{K_{Mapp}^{WT}}{K_{Mapp}^{SNP}}$$

where I represents the presence of an inhibitor, and $K_{M_{app}}$ is the apparent K_M with an inhibitor. However, with our molecular modeling simulations, we are able to compute the ratio of dissociation constants K_I, which allow us to compute the ratio of $K_{M_{app}}$. Finally, after rearranging and substituting for J^{WT+I} in terms of J^{WT}, we are able to express J^{SNP+I} in terms of the ratio of K_I's, K_M's, and the inhibitor concentration $[I]$:

$$J^{SNP+I} = J^{WT} \cdot \frac{K_M^{WT}}{K_M^{SNP}} \cdot \frac{K_I^{SNP}}{K_I^{WT}} \cdot \frac{K_I^{WT}}{K_I^{SNP}+[I]}$$

[Equation E]

Thus, this allows us to simulated a perturbed condition in the presence of different inhibitor concentrations.

Biomarker detection

In order to directly relate the estimated differences in binding free energies to enzyme activity within the model, an estimated level of flux for each enzyme first must be determined under normal physiological conditions of the erythrocyte. To determine the reference flux of each enzyme in the erythrocyte, FVA was run under the constraint that the model must achieve maximal ATP production through the Na^+/K^+ pump. We simulated the actions of the various drug molecules by assuming competitive inhibition of the enzyme, at increasing drug concentrations. The maximal and minimal fluxes through each reaction was then used as a reference rate of reaction for each enzyme.

Markov chain Monte Carlo (MCMC) sampling

Markov chain Monte Carlo sampling was utilized to produce feasible flux distributions within the erythrocyte model [53,54]. A modified version of the artificially centered hit-and-run (ACHR) algorithm was run for COMT and G6PD enzymes. Initial states of wild-type and mutant systems were generated with FVA, and reactions found to be in intracellular loops were discarded from the model before running MCMC sampling. A series of rules were followed to first move each of the flux points randomly while limiting their travel space, and then choosing a new random point along the solution line. Once sampling is complete, each reaction will have a distribution of flux ranges representing the most likely flux for each reaction in the network. Sampling was carried out with the gpSampler module of the COBRA toolbox in MATLAB [55], with the nPoints (number of points) parameter set to 10000.

Results

GEM-PRO coverage

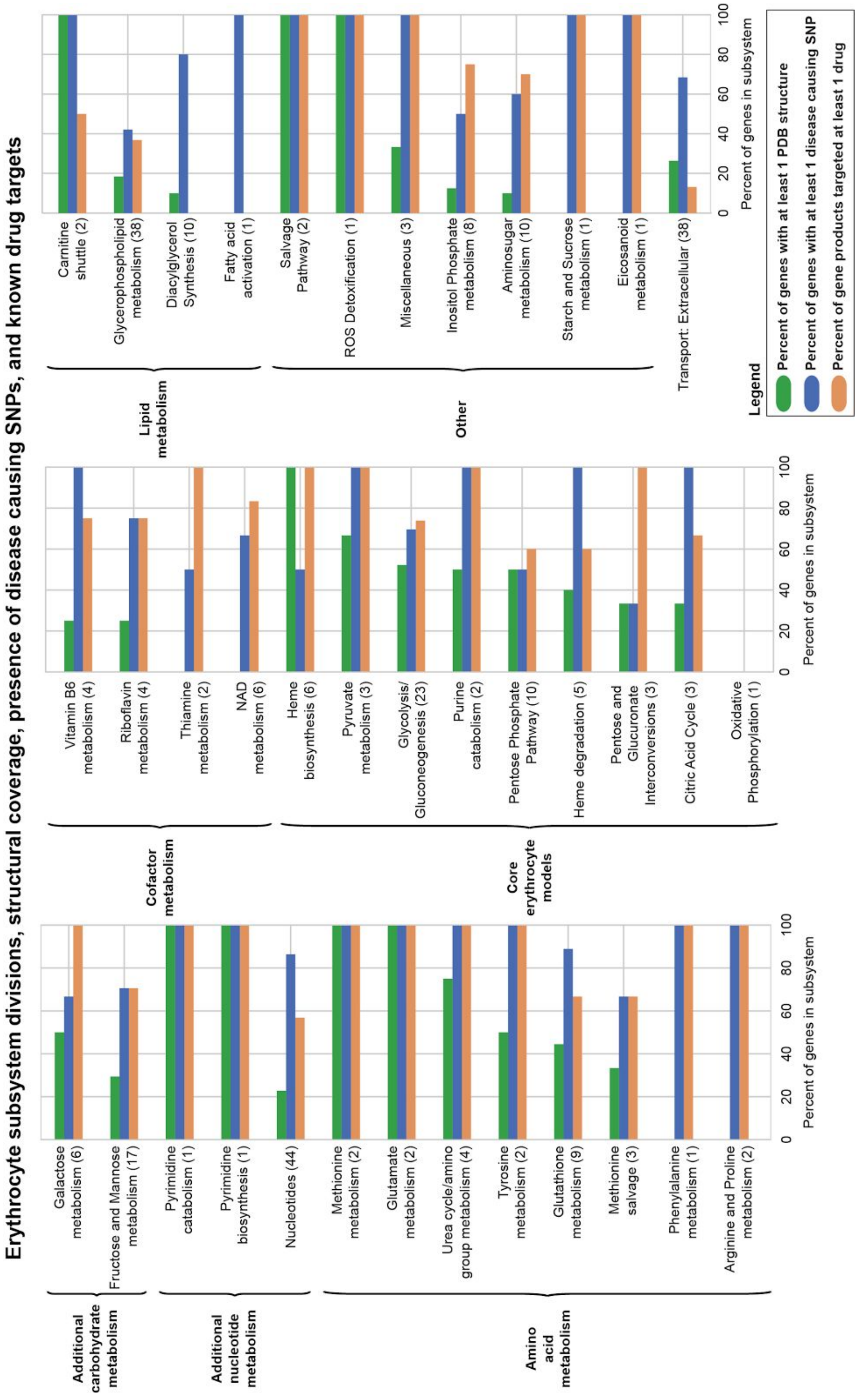

Figure C: Detailed GEM-PRO coverage and pharmacogenomics information per subsystem in the erythrocyte model. Reactions are grouped into general subsystems according to function, and further grouped into smaller subsystems. The number of genes for each smaller subsystem is denoted by the number in parentheses following the text next to each grouped bar. In green are the percent of genes that are represented by an experimental protein structure from the PDB. In blue are the percent of genes with at least one disease causing SNP. In green are the number of genes that have at least one annotated drug targeting it.

Pharmacogenomics data mapping and classification

It is important to note that the drugs identified in this study are significantly associated with an observed adverse reaction but whether the adverse reaction is directly linked to the protein with a particular SNP remains unclear.

Table J: SIFT and PolyPhen predictions of mutations on protein function [32,33].

**Protein**	**UniProt Isoform ID**	**RefSeq ID**	**Ensembl Protein ID**	**dbSNP ID**	**Mutation**	**SIFT score & prediction**	**PolyPhen2 prediction**
COMT	P21964-2	NP_009294	ENSP00000416778	rs4680	Val108Met	0.16 - tolerated	0.222 - benign
G6PD	P11413-1	NP_001035810	ENSP00000377194	rs137852324	Arg454His	0.01 - deleterious	0.975 - damaging
GAPDH	P04406-1	NP_002037	ENSP00000229239	rs11549334	Lys309Asn	0.01 - deleterious	1 - damaging

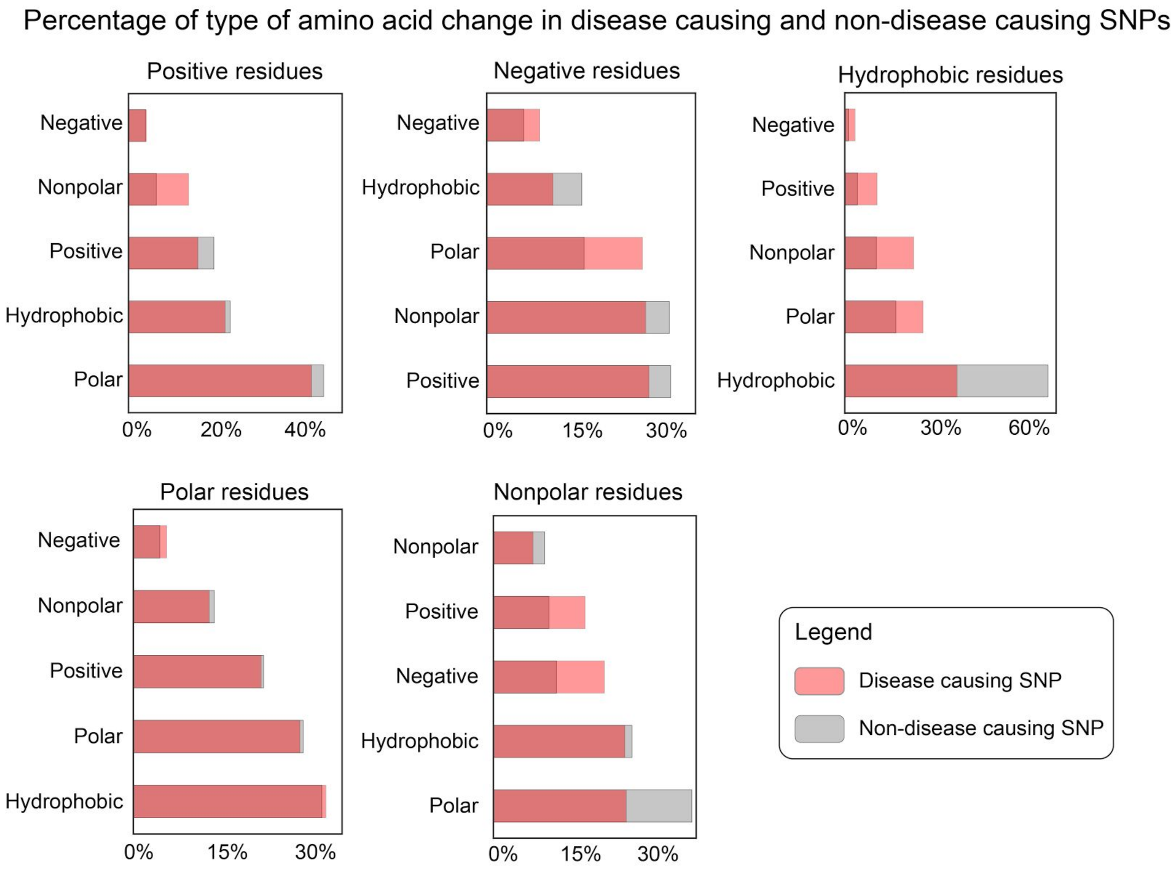

Figure D: Disease causing vs. non-disease causing SNPs. Graphed are the percentages of residues which change from one type of residue to another, as annotated in dbSNP. For instance, on the top right, hydrophobic residues commonly mutate into other hydrophobic residues in non-disease associated SNPs, but in disease causing SNPs, the percentage drastically decreases. Other trends are seen for negative and non-polar residues, while polar residues show no difference.

Molecular modeling simulations

COMT

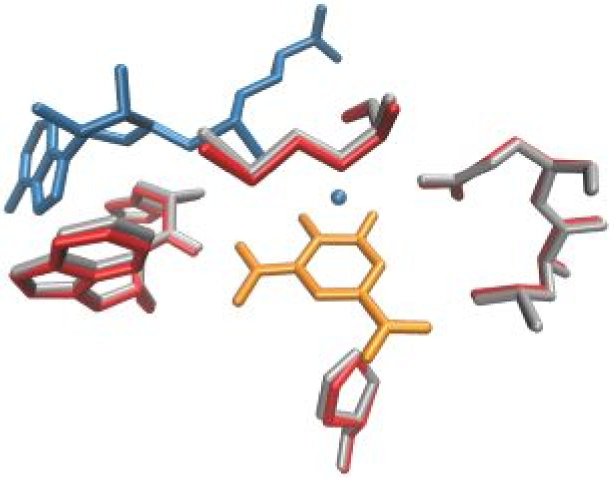

Figure E: Overlay of binding site residues in wild-type (silver, PDB ID: 3BWM) and mutant (red, PDB ID: 3BWY) COMT structures. In blue - cofactor SAM and a magnesium ion. In orange - inhibitor DNC. The crystallized structures align with a 0.2 Å RMSD and are structurally similar.

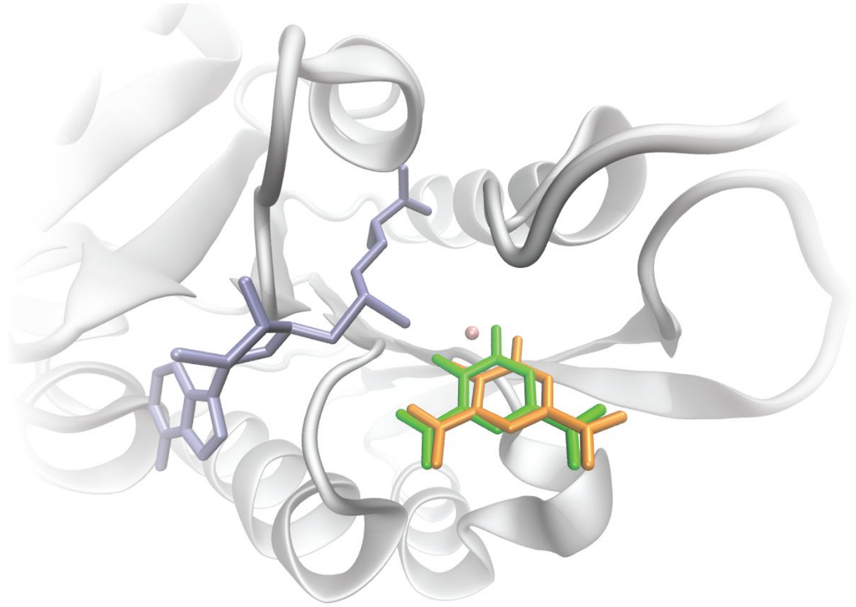

Figure F: Flexible docking of dinitrocatechol (DNC) to wild-type catechol-O-methyltransferase with cofactors. In silver - the protein structure (PDB ID: 3BWM). In light blue is the cofactor S-adenosyl-methionine (SAM), and in pink is a magnesium ion which are required for catalysis. In orange, the original crystallized position of DNC, and in green, the best docked pose of DNC with a distance of < 1 Å RMSD from the original crystallized position.

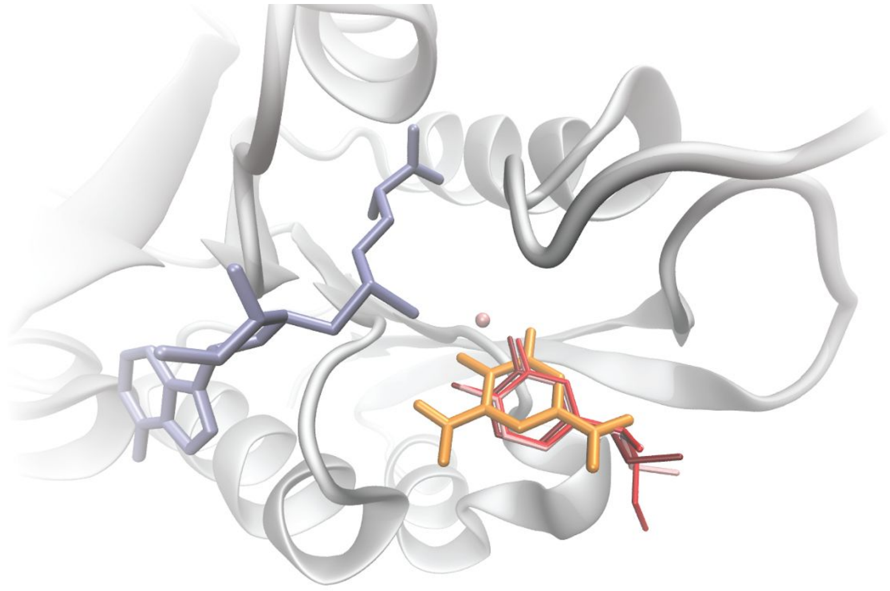

Figure G: Flexible docking of native metabolites dopamine, epinephrine, and norepinephrine, to wild-type catechol-O-methyltransferase with cofactors. In silver - the protein structure (PDB ID: 3BWM). In light blue is the cofactor S-adenosyl-methionine (SAM), and in pink is a magnesium ion which are required for catalysis. In orange, the original crystallized position of dinitrocatechol (DNC). In dark red - the best docked pose of dopamine; red - the best docked pose of epinephrine; pink - the best docked pose of norepinephrine. All native metabolites docked within 2 Å RMSD of equivalent atoms of DNC.

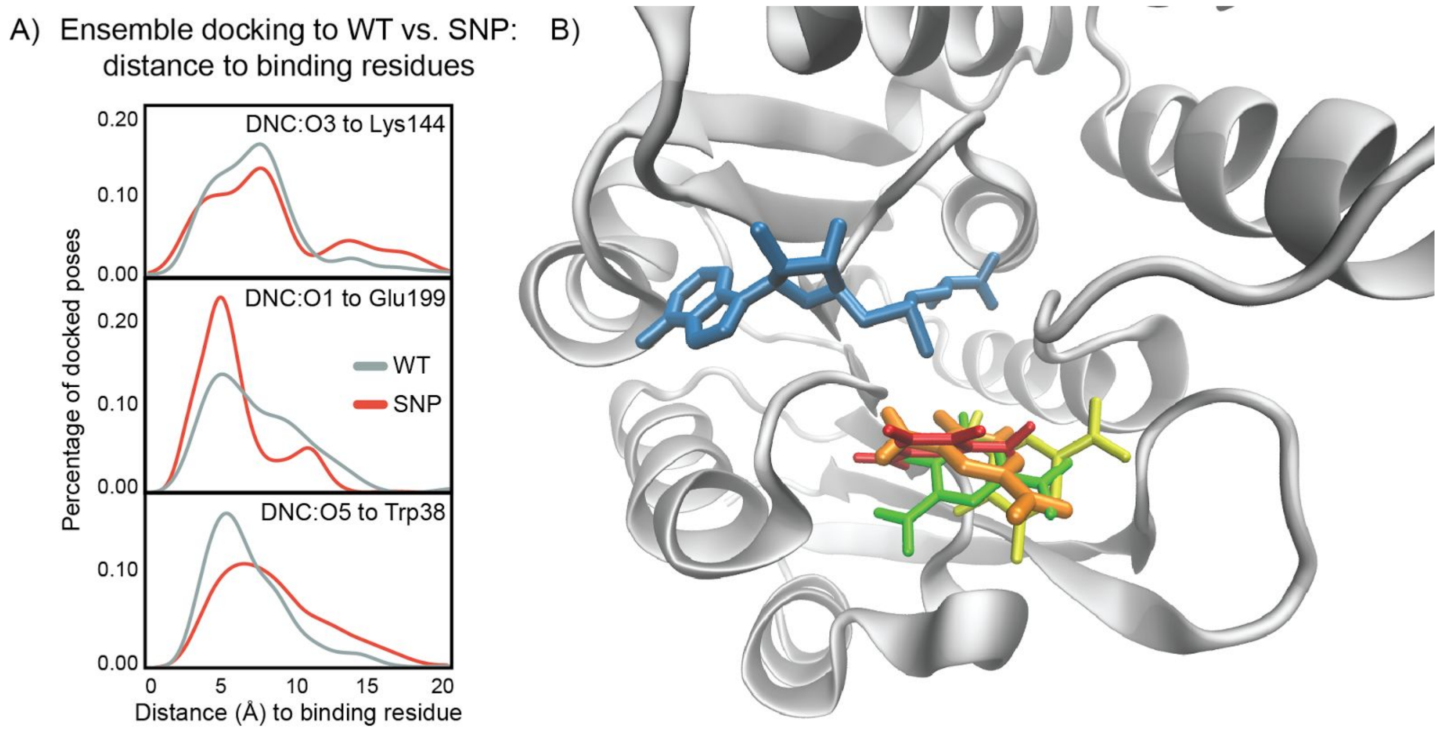

Figure H: A) Distances of the docked ligand (DNC) to binding site residues. Three atom-atom pairs are measured. In silver is the percentage of docked poses in the wild-type ensemble docking results, in red is the SNP ensemble docking results. For two of the three measured distances, the wild-type structure docks more frequently close to the correct binding residues. B) Structural representation of three clusters from the docking distances. In silver - the wild-type protein structure, in blue - the cofactor SAM, in orange - the original position of inhibitor DNC. In green, yellow, and red are the three representative clusterheads from ensemble docking.

Table K: Results from MMPBSA and TI calculations for all metabolites and inhibitors of COMT.

Ligand	Abbreviation	Calculated ΔΔG - MMPBSA	Calculated ΔΔG - TI
Dinitrocatechol	DNC	-7.47 ± 0.66	N/A
Tolcapone	TCW	-4.27 ± 1.15	N/A
Entacapone	ENT	-8.36 ± 1.78	N/A
Dopamine	LDP	-1.77 ± 0.60	-4.251945
Epinephrine	ALE	0.37 ± 0.75	N/A
Norepinephrine	LNR	-3.29 ± 0.85	N/A

G6PD

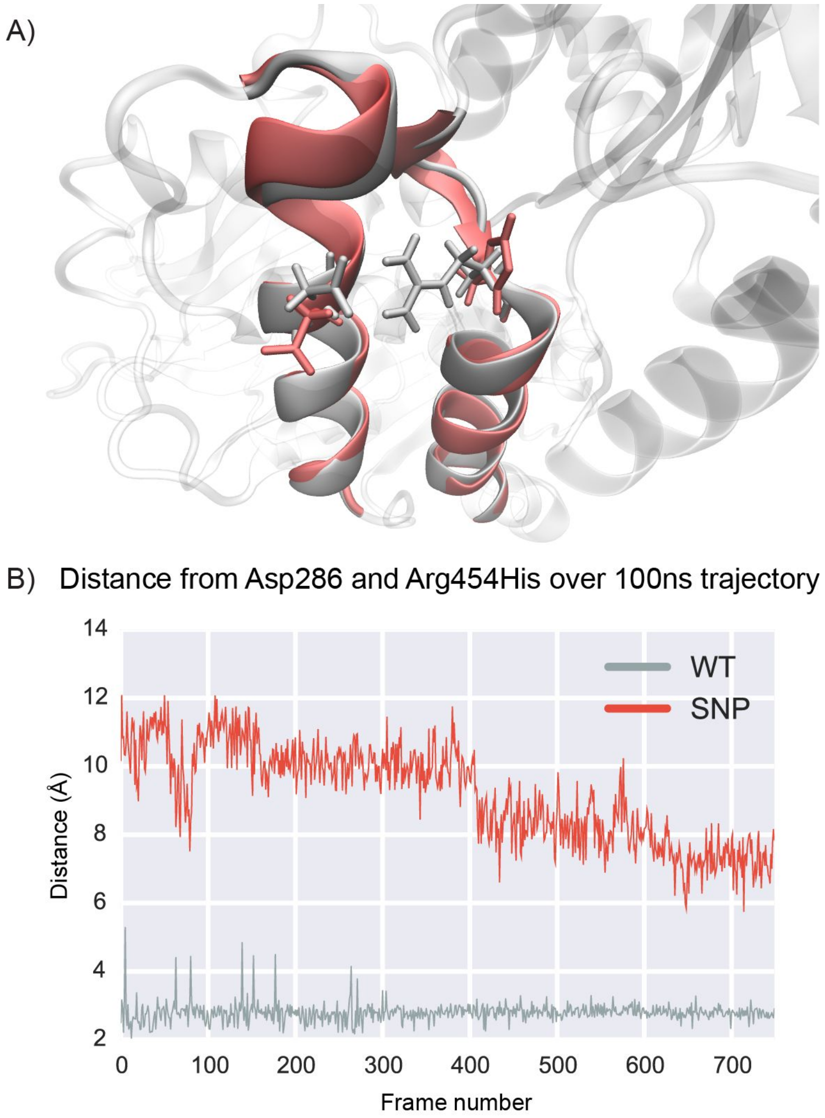

Figure I: Salt bridging interaction of residue 454 to Asp 286. A) Structural view of these two residues in the wild-type (silver) vs. mutant (red) structure. The salt bridge is hypothesized to stabilize the two alpha helices, although through a 100ns MD simulation, little to no changes are observed in the stability or propensity of these alpha helices in the mutant structure. B) plot of the distance between the residues over a 100 ns MD trajectory of the wild-type vs mutant protein. The wild-type protein maintains a salt-bridging distance between the residues while the mutant protein eliminates this.

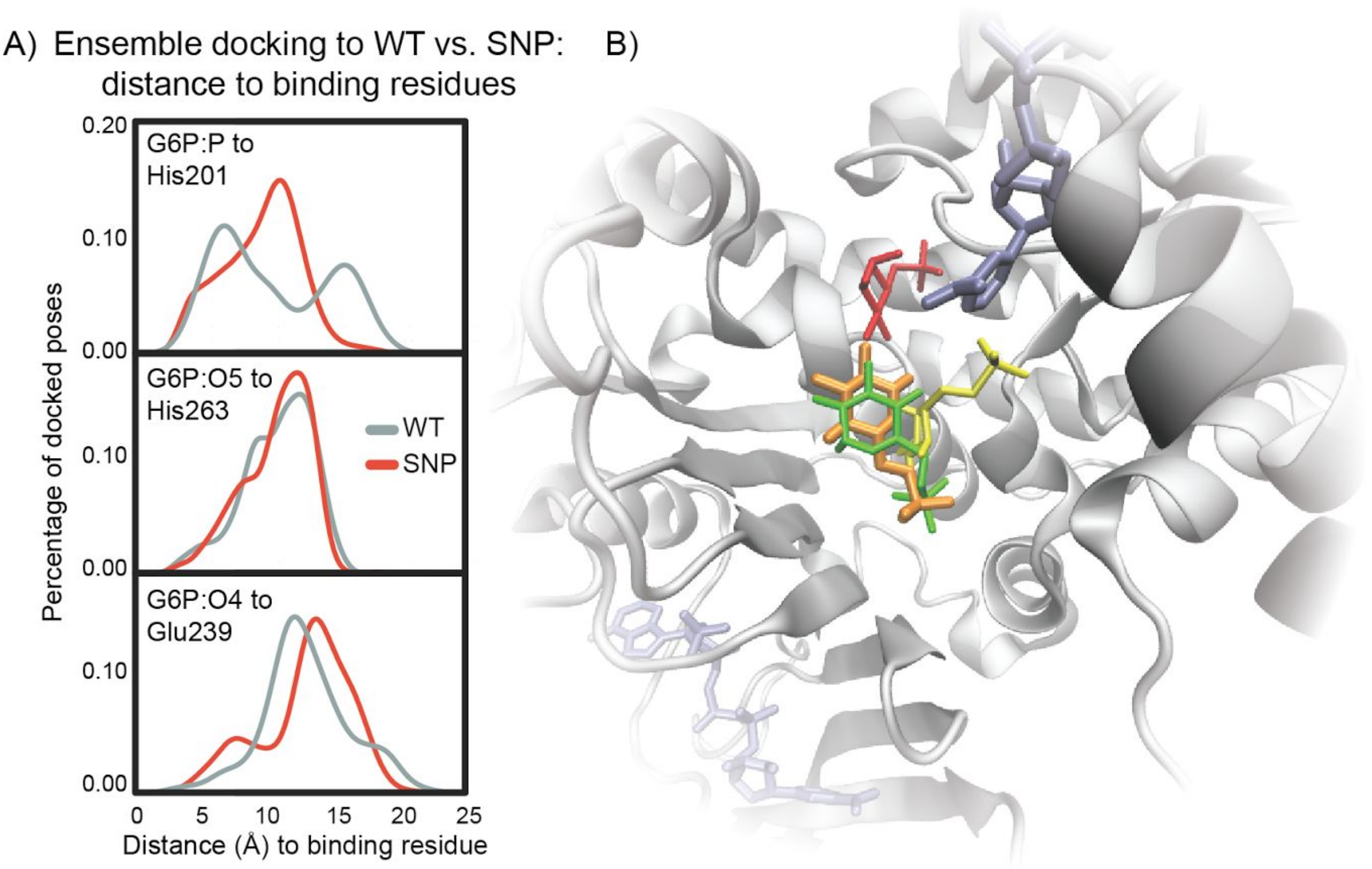

Figure J: A) Distances of the docked ligand (G6P) to binding site residues. Three atom-atom pairs are measured. In silver is the percentage of docked poses in the wild-type ensemble docking results, in red is the SNP ensemble docking results. There was not a discernable difference between these measured distances for wild-type vs. SNP structures. B) Structural representation of three clusters from the docking distances. In silver - the wild-type protein structure, in blue - the cofactor NADP+, in orange - the original position of metabolite G6P. In green, yellow, and red are the three representative clusterheads from ensemble docking.

GAPDH

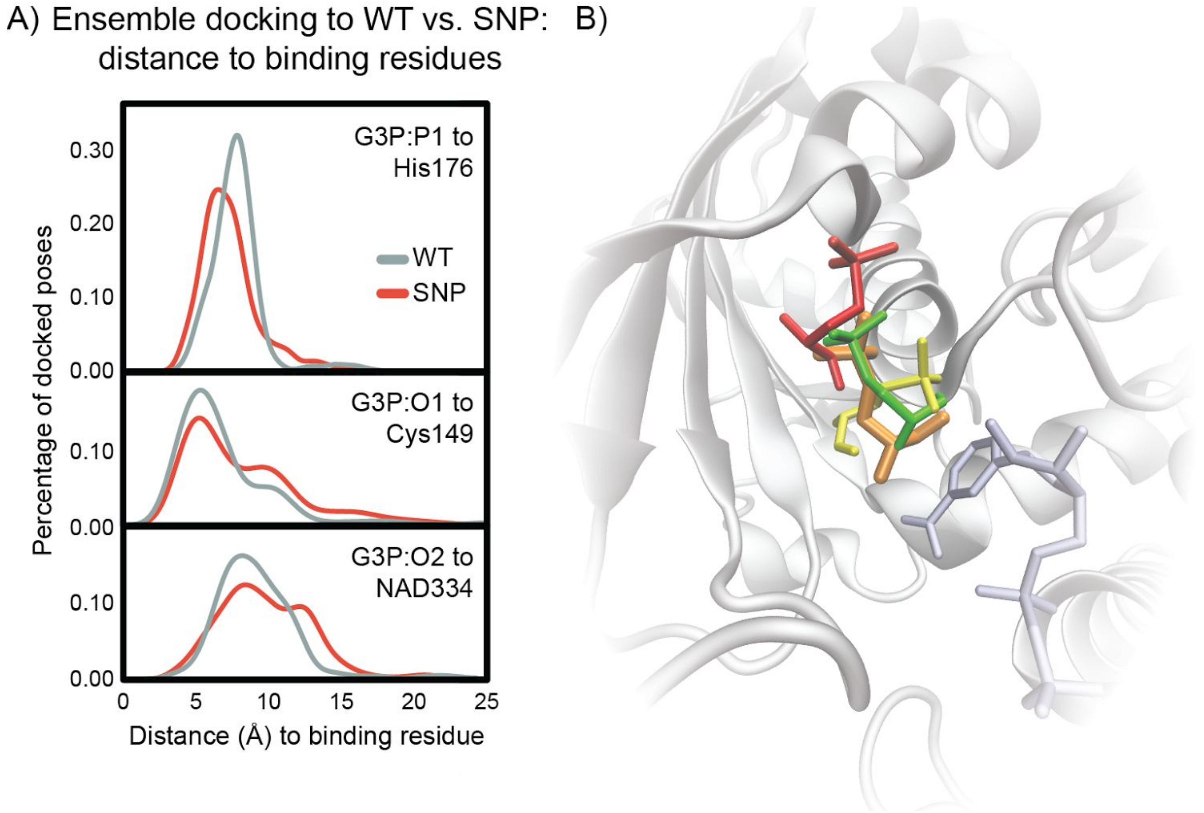

Figure K: A) Distances of the docked ligand (G3P) to binding site residues. Three atom-atom pairs are measured. In silver is the percentage of docked poses in the wild-type ensemble docking results, in red is the SNP ensemble docking results. There was a slight trend for more correct docking in the wild-type structure. B) Structural representation of three clusters from the docking distances. In silver - the wild-type protein structure, in blue - the cofactor NAD+, in orange - the original position of metabolite G3P. In green, yellow, and red are the three representative clusterheads from ensemble docking.

Additional References

1. Zhang Y, Thiele I, Weekes D, Li Z, Jaroszewski L, Ginalski K, et al. Three-dimensional structural view of the central metabolic network of Thermotoga maritima. Science. 2009;325: 1544–1549.

2. Chang RL, Xie L, Xie L, Bourne PE, Palsson BØ. Drug off-target effects predicted using structural analysis in the context of a metabolic network model. PLoS Comput Biol. 2010;6: e1000938.

3. Chang RL, Andrews K, Kim D, Li Z, Godzik A, Palsson BO. Structural systems biology evaluation of metabolic thermotolerance in Escherichia coli. Science. 2013;340: 1220–1223.

4. Chang RL, Xie L, Bourne PE, Palsson BO. Antibacterial mechanisms identified through structural systems pharmacology. BMC Syst Biol. 2013;7: 102.

5. Brunk E, Mih N, Monk J, Zhang Z, O'Brien E, Bliven S, et al. Systems Biology of the Structural Proteome. BMC Syst Biol.

6. Bordbar A, Jamshidi N, Palsson BO. iAB-RBC-283: A proteomically derived knowledge-base of erythrocyte metabolism that can be used to simulate its physiological and patho-physiological states. BMC Syst Biol. 2011;5: 110.

7. Roy A, Kucukural A, Zhang Y. I-TASSER: a unified platform for automated protein structure and function prediction. Nat Protoc. 2010;5: 725–738.

8. Arnold K, Kiefer F, Kopp J, Battey JND, Podvinec M, Westbrook JD, et al. The Protein Model Portal. J Struct Funct Genomics. 2009;10: 1–8.

9. Zhou H, Skolnick J. Template-based protein structure modeling using TASSER(VMT.). Proteins. 2012;80: 352–361.

10. Jaroszewski L, Pawlowski K, Godzik A. Multiple Model Approach: Exploring the Limits of Comparative Modeling. J Mol Med. Springer-Verlag; 4: 294–309.

11. Laskowski RA, MacArthur MW, Moss DS, Thornton JM. PROCHECK: a program to check the stereochemical quality of protein structures. J Appl Crystallogr. International Union of Crystallography; 1993;26: 283–291.

12. Cock PJA, Antao T, Chang JT, Chapman BA, Cox CJ, Dalke A, et al. Biopython: freely available Python tools for computational molecular biology and bioinformatics. Bioinformatics. Oxford Univ Press; 2009;25: 1422–1423.

13. Rice P, Longden I, Bleasby A. EMBOSS: the European Molecular Biology Open Software Suite. Trends Genet. 2000;16: 276–277.

14. Case DA, Darden TA, Cheatham TE III, Simmerling CL, Wang J, Duke RE, et al. AmberTools. University of California, San Francisco, CA; 2008.

15. Ramensky V, Bork P, Sunyaev S. Human non-synonymous SNPs: server and survey. Nucleic Acids Res. 2002;30: 3894–3900.

16. Yuriev E, Holien J, Ramsland PA. Improvements, trends, and new ideas in molecular docking: 2012–2013 in review. J Mol Recognit. 2015;28: 581–604.

17. Karplus M, McCammon JA. Molecular dynamics simulations of biomolecules. Nat Struct Biol. 2002;9:

646–652.

18. van der Kamp MW, Schaeffer RD, Jonsson AL, Scouras AD, Simms AM, Toofanny RD, et al. Dynameomics: a comprehensive database of protein dynamics. Structure. 2010;18: 423–435.

19. Hou T, Wang J, Li Y, Wang W. Assessing the performance of the MM/PBSA and MM/GBSA methods. 1. The accuracy of binding free energy calculations based on molecular dynamics simulations. J Chem Inf Model. 2011;51: 69–82.

20. Gumbart JC, Roux B, Chipot C. Standard binding free energies from computer simulations: What is the best strategy? J Chem Theory Comput. 2013;9: 794–802.

21. Senn HM, Thiel W. QM/MM methods for biomolecular systems. Angew Chem Int Ed Engl. 2009;48: 1198–1229.

22. Bayly CI, Cieplak P, Cornell W, Kollman PA. A well-behaved electrostatic potential based method using charge restraints for deriving atomic charges: the RESP model. J Phys Chem. ACS Publications; 1993;97: 10269–10280.

23. Feng Z, Chen L, Maddula H, Akcan O, Oughtred R, Berman HM, et al. Ligand Depot: a data warehouse for ligands bound to macromolecules. Bioinformatics. Oxford Univ Press; 2004;20: 2153–2155.

24. Bolton EE, Wang Y, Thiessen PA, Bryant SH. Chapter 12 - PubChem: Integrated Platform of Small Molecules and Biological Activities. In: Ralph A. Wheeler and David C. Spellmeyer, editor. Annual Reports in Computational Chemistry. Elsevier; 2008. pp. 217–241.

25. Wang J, Wang W, Kollman PA, Case DA. Antechamber: an accessory software package for molecular mechanical calculations. J Am Chem Soc. 2001;222: U403.

26. Rutherford K, Le Trong I, Stenkamp RE, Parson WW. Crystal structures of human 108V and 108M catechol O-methyltransferase. J Mol Biol. 2008;380: 120–130.

27. Chen J, Lipska BK, Halim N, Ma QD, Matsumoto M, Melhem S, et al. Functional analysis of genetic variation in catechol-O-methyltransferase (COMT): effects on mRNA, protein, and enzyme activity in postmortem human brain. Am J Hum Genet. 2004;75: 807–821.

28. Kotaka M, Gover S, Vandeputte-Rutten L, Au SWN, Lam VMS, Adams MJ. Structural studies of glucose-6-phosphate and NADP+ binding to human glucose-6-phosphate dehydrogenase. Acta Crystallogr D Biol Crystallogr. 2005;61: 495–504.

29. Dolinsky TJ, Czodrowski P, Li H, Nielsen JE, Jensen JH, Klebe G, et al. PDB2PQR: expanding and upgrading automated preparation of biomolecular structures for molecular simulations. Nucleic Acids Res. 2007;35: W522–5.

30. Ryde U. On the role of Glu-68 in alcohol dehydrogenase. Protein Sci. 1995;4: 1124–1132.

31. Jenkins JL, Tanner JJ. High-resolution structure of human D-glyceraldehyde-3-phosphate dehydrogenase. Acta Crystallogr D Biol Crystallogr. 2006;62: 290–301.

32. Adzhubei IA, Schmidt S, Peshkin L, Ramensky VE, Gerasimova A, Bork P, et al. A method and server for predicting damaging missense mutations. Nat Methods. 2010;7: 248–249.

33. Kumar P, Henikoff S, Ng PC. Predicting the effects of coding non-synonymous variants on protein function using the SIFT algorithm. Nat Protoc. 2009;4: 1073–1081.

34. Ross C. Walker †., Melanie M. de Souza ‡., Ian P. Mercer ‡., Ian R. Gould †., David R. Klug* ‡. Large and Fast Relaxations inside a Protein: Calculation and Measurement of Reorganization Energies in Alcohol

Dehydrogenase. J Phys Chem B. 2002;106: 11658–11665.

35. Sirover MA. New insights into an old protein: the functional diversity of mammalian glyceraldehyde-3-phosphate dehydrogenase. Biochim Biophys Acta. 1999;1432: 159–184.

36. Soukri A, Mougin A, Corbier C, Wonacott A, Branlant C, Branlant G. Role of the histidine 176 residue in glyceraldehyde-3-phosphate dehydrogenase as probed by site-directed mutagenesis. Biochemistry. 1989;28: 2586–2592.

37. Cook WJ, Senkovich O, Chattopadhyay D. An unexpected phosphate binding site in glyceraldehyde 3-phosphate dehydrogenase: crystal structures of apo, holo and ternary complex of Cryptosporidium parvum enzyme. BMC Struct Biol. 2009;9: 9.

38. Pettersen EF, Goddard TD, Huang CC, Couch GS, Greenblatt DM, Meng EC, et al. UCSF Chimera--a visualization system for exploratory research and analysis. J Comput Chem. 2004;25: 1605–1612.

39. Wong CF, Kua J, Zhang Y, Straatsma TP, McCammon JA. Molecular docking of balanol to dynamics snapshots of protein kinase A. Proteins. 2005;61: 850–858.

40. Park I-H, Li C. Dynamic ligand-induced-fit simulation via enhanced conformational samplings and ensemble dockings: a survivin example. J Phys Chem B. 2010;114: 5144–5153.

41. Lang PT, Brozell SR, Mukherjee S, Pettersen EF, Meng EC, Thomas V, et al. DOCK 6: Combining techniques to model RNA–small molecule complexes. RNA. 2009;15: 1219–1230.

42. Jones E, Oliphant T, Peterson P, Others. SciPy: Open source scientific tools for Python, 2001--. URL http://www scipy org. 2007;73: 86.

43. Männistö PT, Kaakkola S. Catechol-O-methyltransferase (COMT): biochemistry, molecular biology, pharmacology, and clinical efficacy of the new selective COMT inhibitors. Pharmacol Rev. 1999;51: 593–628.

44. Bautista J, Mason PJ, Luzzatto L. Human glucose-6-phosphate dehydrogenase Lysine 205 is dispensable for substrate binding but essential for catalysis. FEBS Lett. 1995;366: 61–64.

45. Massova I, Kollman* PA. Computational Alanine Scanning To Probe Protein−Protein Interactions: A Novel Approach To Evaluate Binding Free Energies. J Am Chem Soc. 1999;121: 8133–8143.

46. Moreira IS, Fernandes PA, Ramos MJ. Computational alanine scanning mutagenesis—An improved methodological approach. J Comput Chem. Wiley Subscription Services, Inc., A Wiley Company; 2007;28: 644–654.

47. Dehouck Y, Kwasigroch JM, Rooman M, Gilis D. BeAtMuSiC: Prediction of changes in protein-protein binding affinity on mutations. Nucleic Acids Res. 2013;41: W333–9.

48. Li M, Petukh M, Alexov E, Panchenko AR. Predicting the Impact of Missense Mutations on Protein–Protein Binding Affinity. J Chem Theory Comput. 2014;10: 1770–1780.

49. Anand P, Nagarajan D, Mukherjee S, Chandra N. ABS–Scan: In silico alanine scanning mutagenesis for binding site residues in protein–ligand complex. F1000Res. 2014;3. doi:10.12688/f1000research.5165.1

50. Petukh M, Li M, Alexov E. Predicting Binding Free Energy Change Caused by Point Mutations with Knowledge-Modified MM/PBSA Method. PLoS Comput Biol. 2015;11: e1004276.

51. Miller BR III, McGee TD Jr, Swails JM, Homeyer N, Gohlke H, Roitberg AE. MMPBSA. py: an efficient program for end-state free energy calculations. J Chem Theory Comput. ACS Publications; 2012;8:

3314–3321.

52. Borea PA, Varani K, Gessi S, Gilli P, Dalpiaz A. Receptor binding thermodynamics as a tool for linking drug efficacy and affinity. Farmaco. 1998;53: 249–254.

53. Price ND, Schellenberger J, Palsson BO. Uniform sampling of steady-state flux spaces: means to design experiments and to interpret enzymopathies. Biophys J. 2004;87: 2172–2186.

54. Mo ML, Palsson BO, Herrgård MJ. Connecting extracellular metabolomic measurements to intracellular flux states in yeast. BMC Syst Biol. 2009;3: 37.

55. Schellenberger J, Que R, Fleming RMT, Thiele I, Orth JD, Feist AM, et al. Quantitative prediction of cellular metabolism with constraint-based models: the COBRA Toolbox v2. 0. Nat Protoc. Nature Publishing Group; 2011;6: 1290–1307.
